# Enter Mn^IV^–NHC: A Dark Photooxidant with a Long-Lived Charge-Transfer Excited State

**DOI:** 10.1021/jacs.4c08588

**Published:** 2024-08-06

**Authors:** Nidhi Kaul, Eyram Asempa, Juan A. Valdez-Moreira, Jeremy M. Smith, Elena Jakubikova, Leif Hammarström

**Affiliations:** † Department of Chemistry − Ångström Laboratory, Uppsala University, Box 523, SE-75120 Uppsala, Sweden; ‡ Department of Chemistry, North Carolina State University, Raleigh, North Carolina 27695, United States; § Department of Chemistry, Indiana University, 800 East Kirkwood Avenue, Bloomington, Indiana 47405, United States

## Abstract

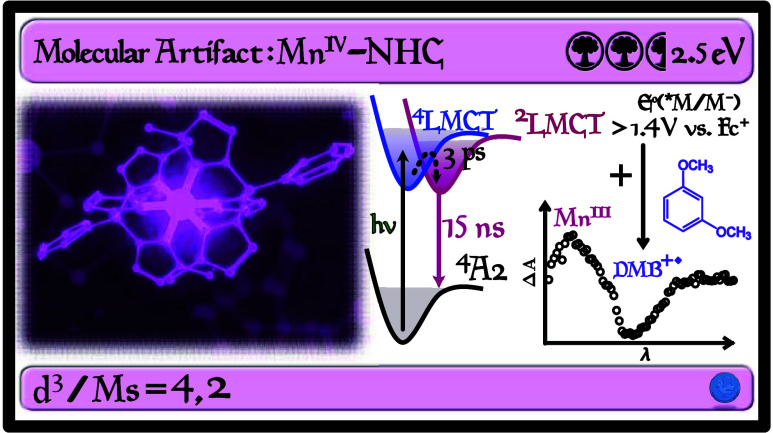

Detailed photophysical investigation of a Mn(IV)-carbene complex has revealed that excitation into its lowest-energy absorption band (∼500 nm) results in the formation of an energetic ligand-to-metal charge-transfer (LMCT) state with a lifetime of 15 ns. To the best of our knowledge, this is the longest lifetime reported for charge-transfer states of first-row-based transition metal complexes in solution, barring those based on Cu, with a d^10^ configuration. A so-called superoxidant, Mn(IV)-carbene exhibits an excited state potential typically only harnessed via excited states of reactive organic radical species. Furthermore, the long-lived excited state in this case is found to be a dark doublet, with its transition to the quartet ground state being spin-forbidden, a contrast to most first-row literature examples, and a possible cause of the long lifetime. Showcasing excited state properties which in some cases exceed those of complexes based on precious metals, these findings not only advance the library of earth-abundant photosensitizers but also shed general insight into the photophysics of d^3^ and related Mn complexes.

## Introduction

Since time immemorial, the central dogma of first-row transition metal complex (TMC) photophysics has been the short-lived nature of its photochemically relevant charge-transfer excited states.^1,2^ At the simplest level, the cause can be traced to the primogenic effect,^3^ where the smaller 3d metals experience an intrinsically lowered ligand field compared to their heavier and larger second- or third-row congeners. Low-lying metal-centered (MC) states thus provide an efficient nonradiative decay pathway for excited states in the charge-transfer (CT) manifold, whose lifetimes typically remain limited to less than 100 fs. The past few years have witnessed a paradigm shift in this dogma, however, and the literature has seen a steady stream of reports on luminescent complexes based on first-row metals, often featuring charge-transfer excited state lifetimes of a few nanoseconds.^4−10^ Key to upending the status quo has been judicious ligand design, which has enabled the realization of large enough field splittings to sufficiently destabilize MC states even in first-row TMCs. In this context, *N*-heterocyclic carbenes (NHCs)—already notable for their steric and electronic properties^11,12^ that have resulted in breakthroughs in organometallic synthesis^13^ and catalysis^14^—have emerged as promising candidates, with their well-known σ-donation assuming central importance.

**Figure 1:**
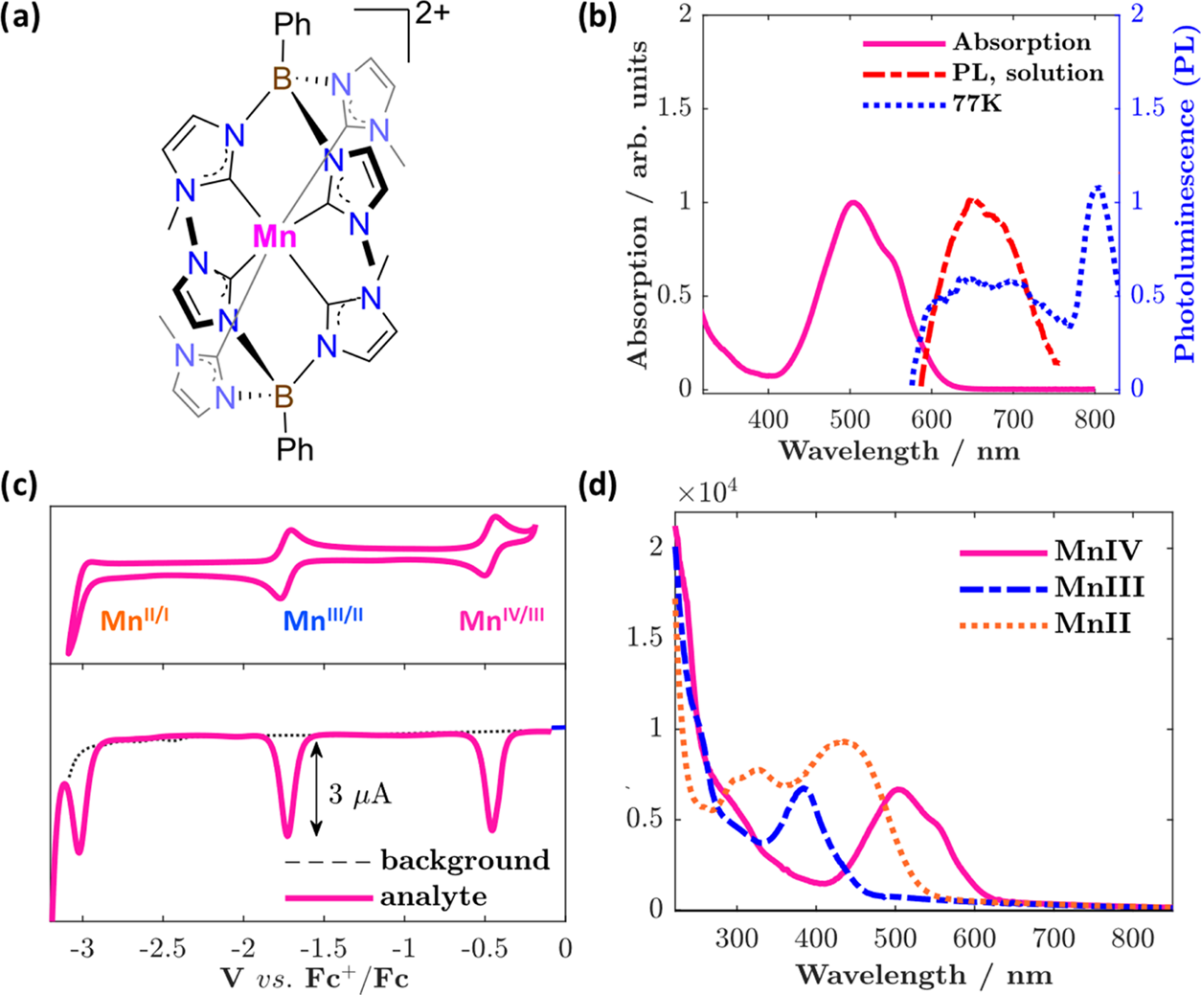
(a) Structure of the complex, [Mn^IV^L_2_]^2+^, under investigation. (b) Normalized steady state absorption (solid pink) and corrected emission spectra of [Mn^IV^L_2_]^2+^ in a ca. 80 μM solution in acetonitrile (dotted red) and at 77 K in butyronitrile glass (dotted blue). λ_ex_ = 500 nm, with excitation and emission slit widths corresponding to a spectral resolution of 5 and 8 nm, respectively. Note that the spectra have been corrected for solvent background, including Raman scatter, for clarity, due to the small emission signals. Uncorrected spectra are supplied in the Supporting Information. (c) Cyclic voltammetry (100 mV/s) and differential pulse voltammetry data for ∼1 mM [Mn^IV^L_2_]^2+^ measured in acetonitrile with 0.1 M TBAPF_6_ as supporting electrolyte. (d) Spectral signatures of the different oxidation states, [Mn^IV^L_2_]^2+^, [Mn^III^L_2_]^+^, and [Mn^II^L_2_]^0^, as determined from controlled potential electrolysis (solid pink, dashed blue, and dotted orange, respectively) on the same solution.

Relatively unique even among NHCs is the tripodal motif,^15−17^ in which *tris*(imidazol-2-ylidene)borates in particular offer a high degree of electronic tunability^18^ and structural integrity. First developed by Fehlhammer in 1996,^19^ a facile new synthetic route was introduced by one of us in 2005,^17^ affording bulkier *tris*(imidazol-2-ylidene)borates able to stabilize low coordinate Fe(II). Later, a phenyl group was introduced on the boron,^20^ resulting in [phenyl(*tris*(3-methylimidazol-1-ylidene))borate]^−^ (L), whose homoleptic Mn(IV) complex, [Mn^IV^L_2_](OTf)_2_, was reported in 2007^21^ (see Figure 1a). Studies carried out a decade later revealed the latter to be the first ever molecular manganese complex exhibiting both CT and metal-centered luminescence at room temperature, albeit in the solid state.^22^ Just a year later in 2018, the Fe(III) analogue was found to possess a doublet ligand-to-metal charge-transfer (^2^LMCT) excited state lifetime of 2 ns in solution at room temperature^23^—regarded, at the time, as record-breaking and prompting the aforementioned flurry of activity.

Surprisingly, the solution phase photophysics of the Mn-based counterpart has never been explored, and we attempt here to plug this lacuna in an effort to gain general insight into the behavior of this subset of complexes. The title compound, [Mn^IV^L_2_]^2+^, possesses a d^3^ electronic configuration, making it a ground state quartet^22^ and isoelectronic with well-known and thoroughly characterized Cr(III) and V(II)-polypyridyl complexes.^24−31^ Consequently, one could anticipate similar photophysics, and such was indeed the case for the solid state, where the metal-centered ^2^E state was found to emit at ∼820 nm, with a lifetime of 1.5 μs. In the solution phase, however, we find a long-lived ^2^LMCT excited state, enriching the rapidly expanding library of photosensitizers based on first-row TMCs. Oftentimes, long-lived CT states are synonymous with luminescence in photophysical parlance: interestingly, however, this 15 ns ^2^LMCT is found to be dark and a potent photooxidant. It is these unusual observations that we seek to elucidate in this report.

## Results and Discussion

### Steady State Spectroscopy

The absorption spectrum of [Mn^IV^L_2_]^2+^ in acetonitrile (Figure 1b, solid pink) features a prominent band in the visible spectrum peaking at 504 nm (∼6200 M^–1^ cm^–1^, Figure 1d) with a shoulder at 552 nm. The potential difference between the Mn^IV/III^ couple and ligand oxidation (*vide infra*) is compatible with the energy of this lowest-energy band; taken together with the magnitude of the extinction coefficient, it is identified as an LMCT transition. Excitation into this charge-transfer band results in a broad, slightly structured emission centered around 660 nm, which in 77 K glass tails off into a sharp band in the near-infrared at ∼820 nm. With a lifetime of 1.5 μs in the solid state at room temperature, the near infrared (NIR) emission has been previously characterized as originating in the ^2^E ligand-field state of the complex^22^ and will not be discussed further here.

The intersection of the normalized LMCT absorption and emission spectra allows for an approximation of the lowest charge-transfer excited state energy, *E*_0–0_, which comes to be approximately ∼2.1 eV. The emission quantum yields are at detection limits (<0.02%), precluding the collection of reliable excitation spectra in solution due to contributions from Raman scatter. In the solid state, however, the excitation spectrum faithfully follows the lowest-energy absorption band (Figure S1) confirming its charge-transfer character. It is worth noting here that the solid state absorption (drop-cast film on a glass substrate) is broadened on the lower-energy side, with the shoulder gaining intensity.

### Electrochemistry and Spectroelectrochemistry

Cyclic and differential pulse voltammograms for the complex are presented in Figure 1c. Two reversible reductions can be seen at *E*_1/2_ = −1.74 and −0.47 V vs Fc^+^/Fc, which can be attributed to the Mn^III/II^ and Mn^IV/III^ couples, respectively. These values are anodically shifted from those previously reported,^21^ but the Mn^III/II^ couple was quasi-reversible under those measurement conditions, possibly due to the presence of oxygen. An occurrence of a redox event at the edge of the electrolyte window can also be noted, precluding its characterization by cyclic voltammetry, but it is easily resolved in the differential pulse voltammogram (−3.02 V). This is likely the Mn^II/I^ couple, keeping in mind that the carbene ligand reduction can be expected to lie further beyond (at least < −3.5 V) based on measurements on previous carbene analogues.^23,32^ It is remarkable that three reversible metal-centered redox events occur within the solvent window. For [MnN_6_]^*n*+^ style complexes, extant series^33,34^ are those of [Mn(tpy)_2_]^*n*+^ (tpy = terpyridine) and [Mn(dgpy)_2_]^*n*+^ (dgpy = 2,6-diguanidylpyridine), with the latter only recently fully characterized and reported by Heinze and co-workers.^34^ The Mn^IV/III^ couple potentials are +1.39 and +0.58 V, respectively, see Table 1. For the related tripodal trispyrazolyl-borate Mn(IV) complex, the Mn^IV/III^ redox event occurs at nearly 1 V, producing a powerful ground state oxidant. This is in sharp contrast to the current complex whose oxidizing ability is manifested in the excited state, owing to the substantial lowering of couple potentials due to the carbene donors.

**Table 1:** Electrochemical Potentials^a^ and the Calculated^b^ Ligand-Field Splitting Parameter (Δ_o_ = 10 Dq) for [Mn^IV^L_2_]^2+^ and [MnN_6_]^*n*+^ Complexes^c^

	M^IV/III^	M^III/II^	Mn^II/I^	Δ_o_ = 10 Dq (eV)^b^
[Mn^IV^L_2_]^2+^	–0.47	–1.74	–3.02^d^	8.25
[Mn^II^(dgpy)_2_]^(2+c)^	0.58	–0.26	–	7.84
[Mn^II^(tpy)_2_]^(2+c)^	1.39	0.88	–	7.18

a Referenced vs the Fc^+^/Fc couple.

b Estimated using TD-DFT calculations for the Mn^IV^ oxidation state, see below.

c Electrochemical data from refs (33) and (34) for [Mn^II^(tpy)_2_]^2+^ and [Mn^II^(dgpy)_2_]^2+^, respectively.

d Peak potential from DPV.

The Mn^IV/III^ couple potential taken together with the above-determined *E*_0–0_ of 2.1 eV enables a first approximation of the excited state reduction potential, *E*^0^(*M/M^–^) ≈ 1.63 V (or ca. 2 V vs SCE). The latter value is comparable to those reported for organic radical excited states^35,36^—dubbed as “superoxidants”—and indeed there is evidence that the complex is able to oxidize several organic substrates (see the Reactivity section). This behavior is analogous to that seen in complexes of heavier congeners Tc and Re with the donor ligand dmpe (dmpe = bis-1,2-(dimethylphosphino)ethane).^37,38^ Their fluorescent ^2^LMCT excited states have potentials of up to +2.22 V (+2.6 vs SCE), which are some of the highest ever reported for simple mononuclear complexes of transition metals. These specific cases notwithstanding, Mn(IV)-carbene presents a sizable improvement in the accessible potential over a slew of oft-employed precious metal photosensitizers.^39−41^ It also presents enhancement of metrics such as extinction coefficient or lifetime compared to its predecessor Fe(III)-carbene, and several other first-row-based metal complexes, including the recently reported^42^ [Mn^IV^(dgpy)_2_]^4+^. Some representative examples are collected in Table 2 for a ready reference.

**Table 2:** Key Photophysical Parameters for [Mn^IV^L_2_]^2+^ and Some Relevant Photosensitizers Reported in the Literature^a^

complex, excited state	ε_max_ (M^–1^ cm^–1^)	*E*^0^(*M/M^–^)^b^	lifetime	refs
[Mn^IV^L_2_]^2+^, ^2^LMCT	504 nm, 6200	>1.5^c^	15 ns	this work
[Mn^IV^(dgpy)_2_]^4+^, ^2^LMCT	514 nm, 6860	1.42	1.6 ns	(42)
[Fe^III^L_2_]^+^, ^2^LMCT	502 nm, 3000	0.98	2 ns	(23)
[Re(dmpe)_3_]^2+^, ^2^LMCT	528 nm, 2110	2.23	11 ns	(37,38)
[Ru(bpy)_3_]^2+^, ^3^MLCT	452 nm, 14,600	0.46	1.1 μs^d^	(39)
[Ir[dF(CF_3_)ppy]_2_(dtbpy)]^+^, ^3^MLCT	380 nm, 6170	0.83	2.3 μs^d^	(41)

a Note that a high emission quantum yield is not necessary for photosensitizer efficacy, though it may be otherwise desirable for different applications.

b vs the Fc^+^/Fc couple. Potentials reported vs SCE in the literature were converted by subtracting 380 mV.

c See the Reactivity section for details.

d Note these are values in deaerated solvent; the ^2^LMCT excited states are not quenched by oxygen, and may offer advantages in certain contexts over the typically utilized ^3^MLCT excited states.

The impressive excited state potential directly translates from the lowering of the Mn^IV/III^ couple potential by over 700 mV as compared to the iron analogue, Fe^IV/III^ = 0.25(5) V, which possesses a similar *E*_0–0_ of 2.1 eV.^23^ While a direct cause cannot be delineated, some informed comparisons are insightful. Generally, M^*n*+/*n*^ potentials increase in the first row from left to right, and the observation is consistent with such a trend. Similar behavior has been seen for isostructural *tris*(pyrazolyl)borate (Tp) complexes for M^4+/3+^ couples (the Fe^IV/III^ couple for [Fe(Tp)_2_] is accessible only in liquid sulfur dioxide;^43^ a comparison suggests a 300 mV difference, with Mn easier to oxidize^44^). It is notable that, with Tp being a relatively weaker field ligand, the trend is reversed for M^3+/2+^ couples, possibly due to spin-pairing effects. In that regard, the observations here are consistent with expected trends, and the more pronounced difference may be a simple consequence of the much stronger σ-donation of the *tris*(carbene)borates compared with Tp ligands. On the other hand, the observation in this case could result, in part, due to a difference in the size of the metal center (Mn > Fe), which allows for better orbital overlap of the ligand with Mn. This notion finds a degree of support in the twice as large extinction coefficient observed for the LMCT transition in the Mn complex compared to the Fe.

Spectroelectrochemistry allows for further confirmation to the ground state absorption assignment, where controlled potential electrolysis at −0.8 V results in bleaching of the LMCT band (Figure S6) and the growth of a new band centered at 384 nm (ε ≈ 6300 M^–1^ cm^–1^) from the resulting [Mn^III^L_2_]^+^ complex (Figure 1d, dashed blue), in good agreement with that reported prior.^45^ This too is most reasonably interpreted as an LMCT transition based on the energy of ca. 3.5 eV, which is consistent with a potential difference of the Mn^III/II^ couple and the ligand oxidation (Figure S5). Lastly, further reduction at −2 V (Figure S6) produces [Mn^II^L_2_], whose broad absorption consists of a maximum at 436 nm (ε ≈ 8870 M^–1^ cm^–1^), together with higher-energy peaks at 326 and 300 nm (Figure 1d, orange). An MLCT assignment of the lowest-energy band at 436 nm would place the carbene ligand reduction below −4.5 V, which is consistent with previous estimates.^23^ These diagnostic spectral traces will aid in the interpretation of the transient absorption data.

### Time-Dependent Density Functional Theory (TD-DFT) Calculations

**Figure 2:**
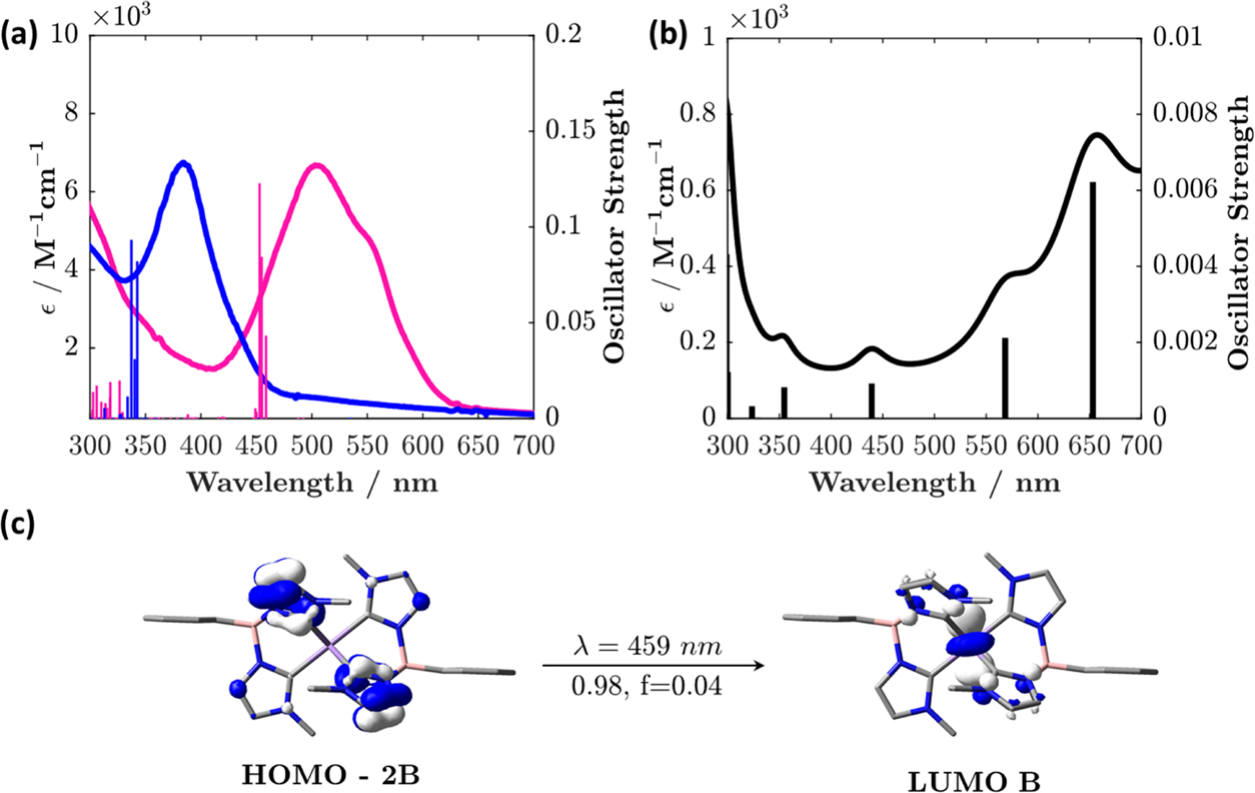
(a) Experimental absorption spectrum with the corresponding calculated stick spectrum of [Mn^IV^L_2_]^2+^ (pink) and [Mn^III^L_2_]^+^ (blue). (b) Calculated absorption spectrum and the corresponding stick spectrum of [Zn^II^(L)Cl]^+^. (c) Molecular orbitals for the calculated lowest-energy transition (λ = 459 nm) in [Mn^IV^L_2_]^2+^. B represents “β”. *f* is the oscillator strength, mentioned together with the TD-DFT coefficient, below the arrow (see the SI for all transitions).

The LMCT nature of the lowest-energy band in [Mn^IV^L_2_]^2+^ can be further corroborated by simulating its electronic structure and absorption spectra by using DFT and TD-DFT methods. Despite the ultraviolet–visible (UV–vis) calculations being challenging for open-shell systems, good qualitative agreement is obtained between the computed transitions and those observed experimentally; see Figure 2. The experimental absorption spectrum of [Mn^IV^L_2_]^2+^ along with the corresponding calculated stick spectrum is shown in Figure 2a. The calculated stick spectrum features a cluster of transitions around 450 nm (corresponding to the lower-energy peak at ∼500 nm in the experimental spectrum) and another set of transitions centered at ca. 300 nm (corresponding to the shoulder at ∼300 nm in the experimental spectrum; see Figure 2a). The three most intense transitions in the lowest-energy band (at 459, 455, and 453 nm) were determined to be LMCT in their character (Figure 2c and Table S1). The LMCT assignment of the singly reduced species, [Mn^III^L_2_]^+^, and its resultant absorption spectrum can also be confirmed (Figure 2a, blue, Figure S22 and Table S2). Diagnostic features for the oxidized carbene ligand could not be determined using spectroelectrochemistry, owing to the redox event occurring at the edge of the solvent window; indeed, such data has proved elusive in related past publications.^23,46,47^

Thus, in order to simulate the oxidized ligand’s spectrum with reasonable accuracy, geometry optimization and calculations were performed on a model [Zn^II^(L)Cl]^0^ complex, where the metal can be expected to be redox-inactive. The spectrum of the [Zn^II^(L)Cl]^0^ complex shows appreciable absorption only below <250 nm, which is where carbene absorption can be expected. The computed [Zn^II^(L)Cl]^0^ complex was then oxidized to [Zn^II^(L)Cl]^+^, where the electron was taken from the ligand L. The structure of [Zn^II^(L)Cl]^+^ was not reoptimized after oxidation to prevent ligand detachment. The results—plotted in Figure 2b—indicate that the oxidized carbene ligand possesses broad absorption features in the red, above 550 nm, with transitions occurring on the oxidized carbene ligand. The calculated absorption spectra of [Zn^II^(L)Cl]^0^ and the oxidized [Zn^II^(L)Cl]^+^ with their transition assignments are shown in Figures S23–S24 and Tables S3–S4.

As mentioned in the Introduction section, low-lying metal-centered states serve as efficient sinks for CT states in first-row-based TMCs. Pursuantly, the ligand-field splitting parameter, Δ_o_ (=10 Dq), assumes critical importance to ascertain their destabilization. Experimental determination of Δ_o_ is unfortunately not possible in this case: as for many other TMCs, the weak d–d bands are obscured by much more intense charge-transfer transitions. Nevertheless, a computational analysis of the 10 Dq value in this and related Mn complexes to obtain a trend should prove instructive. The results obtained using TD-DFT for [Mn^IV^L_2_]^2+^, [Mn^IV^(dgpy)_2_]^4+^, and [Mn^IV^(tpy)_2_]^4+^ are shown in Table 1, and it is found that the 10 Dq varies as [Mn^IV^L_2_]^2+^ (8.25 eV) > [Mn^IV^(dgpy)_2_]^4+^ (7.84 eV) > [Mn^IV^(tpy)_2_]^4+^ (7.18 eV). Note that due to the large mixing of the MC and LMCT/MLCT transitions in these complexes, the absolute values from TD-DFT calculations are not reliable.

The obtained absolute magnitude of 8.25 eV (over 65,000 cm^–1^) suggests metal-centered transitions around 150 nm and is likely too large. It is possible to refine this estimate by careful consideration of closely related complexes. The cobalt analogue, [Co^III^L_2_]^+^, has an experimentally determined 10 Dq of nearly 4.8 eV (38,500 cm^–1^). A previous attempt^45^ has also been made to measure [Mn^III^L_2_]^+^, but was thwarted by facile oxidation to [Mn^IV^L_2_]^2+^, the complex under investigation herein. Related Mn(III)-pyrazolyl borates could be measured using MCD, however, with determined 10 Dq values in the range 20–25,000 cm^–1^. Calculations at the CASSCF/NEVPT2 level of theory^48^ could reproduce these numbers fairly well, and this same method applied to [Mn^III^L_2_]^+^ suggested a 10 Dq of over 35,000 cm^–1^. It stands to reason that a larger value may be suspected for Mn(IV)-carbene, owing to the higher charge on the metal center. Furthermore, comparing the metal couple potentials as a proxy for improved ligand–metal overlap, one might consider that Δ_o_ should vary as [Co^III^L_2_]^+^ < [Fe^III^L_2_]^+^ < [Mn^IV^L_2_]^2+^, congruent with the M^4+/3+^ reduction potentials observed in these complexes.^23,32^ Collectively, this line of arguments suggests a 10 Dq value in excess of 40,000 cm^–1^ as a reasonably conservative estimate, putatively pointing to one of the largest Δ_o_ values accessible in first-row-based TMCs.

### Time-Resolved Spectroscopy

**Figure 3:**
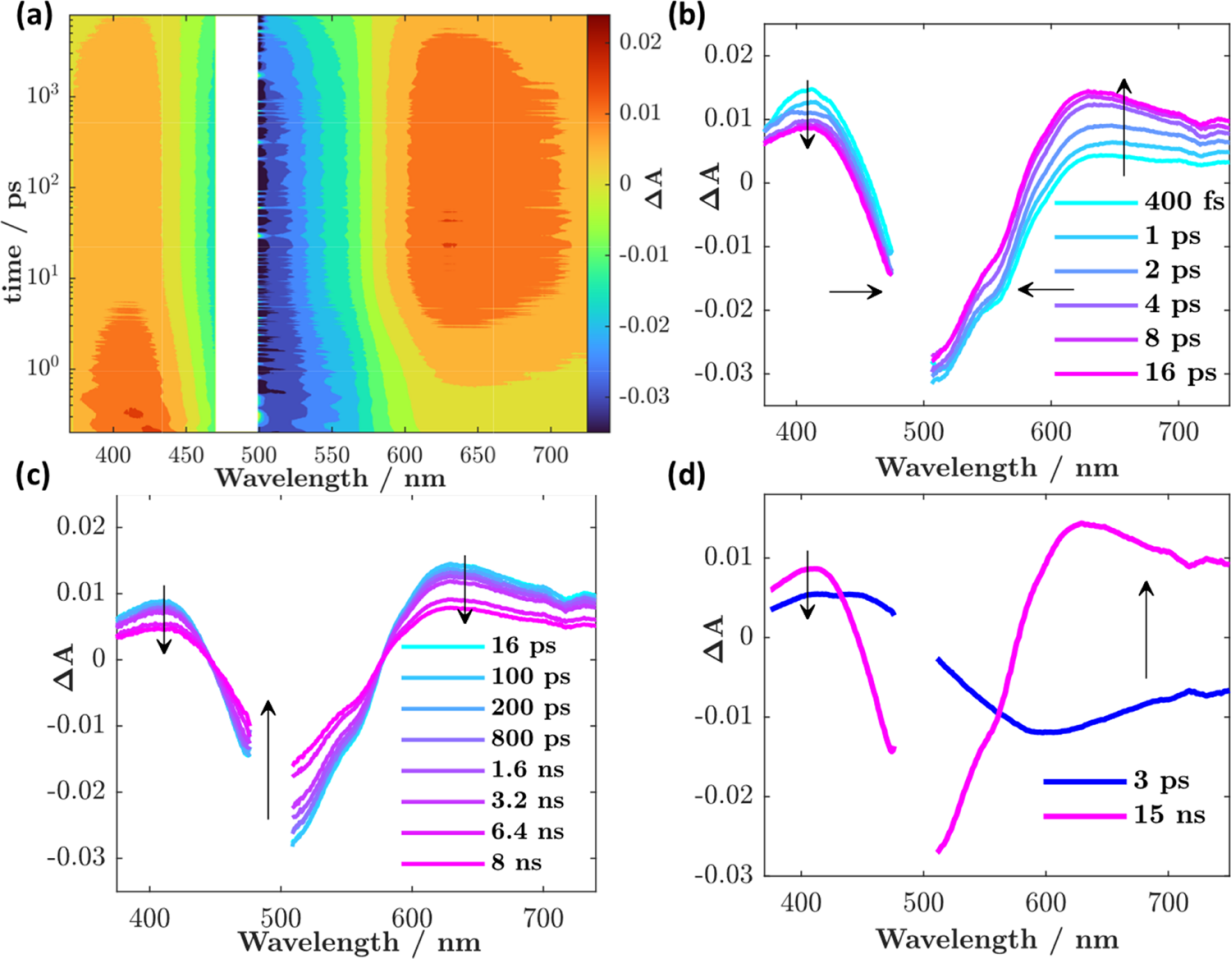
(a) Contour map of femtosecond transient absorption data recorded for [Mn^IV^L_2_]^2+^ in acetonitrile (excitation wavelength = 480 nm, pulse energy = 0.7 μJ/pulse, absorption ≈0.35 at the excitation wavelength). (b, c) Spectral traces at selected time points (horizontal cuts from (a) with arrows to guide the spectral evolution. (d) Decay-associated spectra obtained from a global analysis of the data in (a).

Insight into the excited state dynamics of [Mn^IV^L_2_]^2+^ can be gleaned via femtosecond transient absorption spectroscopy. Upon excitation at 480 nm into the lowest-energy band, the overall spectral shape at all time scales (Figure 3a) is consistent with excited state absorption signals in the blue due to the transiently formed Mn^III^ (Figure 1d) in accordance with the LMCT nature of the transition, together with the expected Mn^IV^ ground state bleach centered at around 500 nm. Although they were unable to be determined using spectroelectrochemistry, spectra obtained for the oxidized ligand using TD-DFT (*vide supra*) suggest a broad absorption at wavelengths greater than 550 nm. Consequently, positive features seen toward the red at later time scales likely stem from the oxidized ligand itself—as seen in previous carbene analogues—or transitions to it from the transiently reduced metal. In the initial time scales (<3 ps), stimulated emission (>600 nm) is superposed on the positive features of the oxidized ligand, see below. In totality, the observations support a predominantly charge-transfer assignment at all observed time scales.

A global analysis of the transient data reveals biexponential decay behavior: a 3 ps component representative of excited state conversion, and a longer 15 ns component reflecting the complete ground state recovery; the relevant decay-associated spectra are plotted in Figure 3d, and kinetics with global fits can be found in Figure S8. Bearing in mind that the accessible time window in the femtosecond experiment is 8 ns, i.e., ca. one-half-life of the determined time constant, the residuals matrix was carefully analyzed to determine the best fit (see the Supporting Information), yielding τ = 15 ± 1 ns. The complete and single-exponential ground state recovery with the same τ = 15 ± 1 ns could also be monitored using flash photolysis (Figure 5a,b) and the transient spectrum was found in excellent agreement with the femtosecond transient absorption data. Furthermore, no detectable changes could be observed in sample absorption before and after measurements (Figure S10), confirming the photostability of the complex.

The initial spectral evolution in the first few picoseconds is characterized by a narrowing of the bleach band centered at ∼500 nm and a concomitant growth in signal toward the red (Figure 3b). The latter occurs where stimulated emission signals from the LMCT excited state can be expected (Figure 1b), and it is best interpreted as a loss thereof. A simultaneous decrease in amplitude of the excited state absorption in the blue, peaking at 409 nm, can also be observed.

Making strict state assignments based on transient data alone is challenging for TMCs. The traditionally assumed paradigm of *k*_vib_ > *k*_ic_ > *k*_isc_ from organic photochemistry does *not* necessarily hold for them,^49^ because they possess a plethora of excited state manifolds.^2,50^ That being said, in this case, it is reasonable to assume that the initially formed Franck–Condon state has quartet multiplicity given the ^4^A_2_ ground state. A clear loss of stimulated emission intensity (Figure 3b,d) in the earlier time scale data supports the fact that the initially observed excited state is the ^4^LMCT for this complex, which proceeds to disappear in around 3 ps. A calculation using the Strickler–Berg equation—well suited to treat symmetry and spin-allowed transitions—may be made for order-of-magnitude estimates
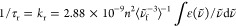
1
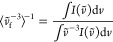
2Here, *n* is the solvent refractive index, *I* is the emission intensity, ε is the extinction coefficient in M^–1^ cm^–1^, and *v ~* is wavenumber in cm^–1^. The relevant integrated absorption and emission spectra are 3.4 × 10^12^ cm^3^ and 1.3 × 10^3^ cm^–4^ and together with *n* = 1.34 for acetonitrile, yield a radiative rate constant, *k*_r_, of 2.4 × 10^7^ s^–1^ (radiative lifetime ). The observed lifetime, τ_obs_, may thus be evaluated by using ϕ_em_ = *k*_r_τ_obs_. Plugging in the detection limit emission quantum yield of 10^–4^ gives an excited state lifetime of 4.2 ps, which is in very good agreement with the 3 ps observed experimentally. The observed emission can therefore be reasonably ascribed to the initially populated, short-lived ^4^LMCT state.

This short component could, in principle, represent intersystem crossing to the ^2^LMCT state or to the ligand-field manifold in competition with vibrational cooling. Direct population of the ^2^E state from the ^4^LMCT has been argued to occur on a time scale of ∼50 fs in isoelectronic Cr(acac)_3_^49^—if this system was considered in analogy, with the state observed in Figure 3c being the ^2^E, notable contradictions occur. (1) The ^2^E state is too low in energy to explain the observed electron transfer reactivity (*vide infra*), (2) the observed transients retain primarily charge-transfer contributions, which is at odds with an assignment to a pure ligand field state, and (3) the observed lifetime of 15 ns would be unusually small for the minimally distorted, spin-flip, ^2^E state. Lifetimes in the microsecond regime are routine in related d^3^ Cr complexes,^24,25,28,51−54^ and even [MnL_2_]^2+^ in the solid state features a 1.5 μs long-lived ^2^E state.^22^ Finally, a change to the doublet multiplicity within the LMCT manifold is not only consistent with the relatively subtle spectral changes but would also make the transition back to the ground state spin-forbidden. The latter could help explain the nearly order of magnitude longer lifetime for the LMCT excited state of this complex compared to its [Fe^III^L_2_]^+^ analogue, and others, which exhibit spin-allowed decays.

These arguments suggest that the long-lived component can be identified as a long-lived dark ^2^LMCT state, which is exceedingly rare. It can be noted that intersystem crossing from the ^4^LMCT to ^2^LMCT is a violation of Hund’s rule of maximum multiplicity, but such exceptions have been seen in related complexes,^31,55^ with antiferromagnetic coupling being implicated. In this complex, TD-DFT calculations indicate a range of geometries at which ^4^LMCT and ^2^LMCT are nearly isoenergetic, with ^2^LMCT being lower in energy as well (Figure S25). Consequently, the observed intersystem crossing event is indeed permissible in this complex. A dynamic Jahn–Teller effect could also be thought to be operative in this instance; depending on the hole occupancy, two of the three t_2g_ orbitals can be thought to be preferentially stabilized in the crystal field perspective, lifting the degeneracy and resulting in their full occupancy in the excited state (which is a d^4^), and radical character on the ligand. The initially observed 3 ps ^4^LMCT lifetime allows for calculation of an upper limit of the intersystem crossing rate constant (*k*_isc_) as the observed rate constant, *k*_obs_ = 1/τ_obs_ = *k*_r_ + *k*_nr_ + *k*_isc_ ≤ 3.3 × 10^11^ s^–1^. Even in the limiting case where *k*_isc_ ≈ *k*_obs_, it would still be substantially slower than that typically observed for isoelectronic Cr and V complexes (>10^12^ s^–1^). Curiously, it is similar to intersystem crossing rates seen in Mn-porphyrins, however.^56^

**Figure 4:**
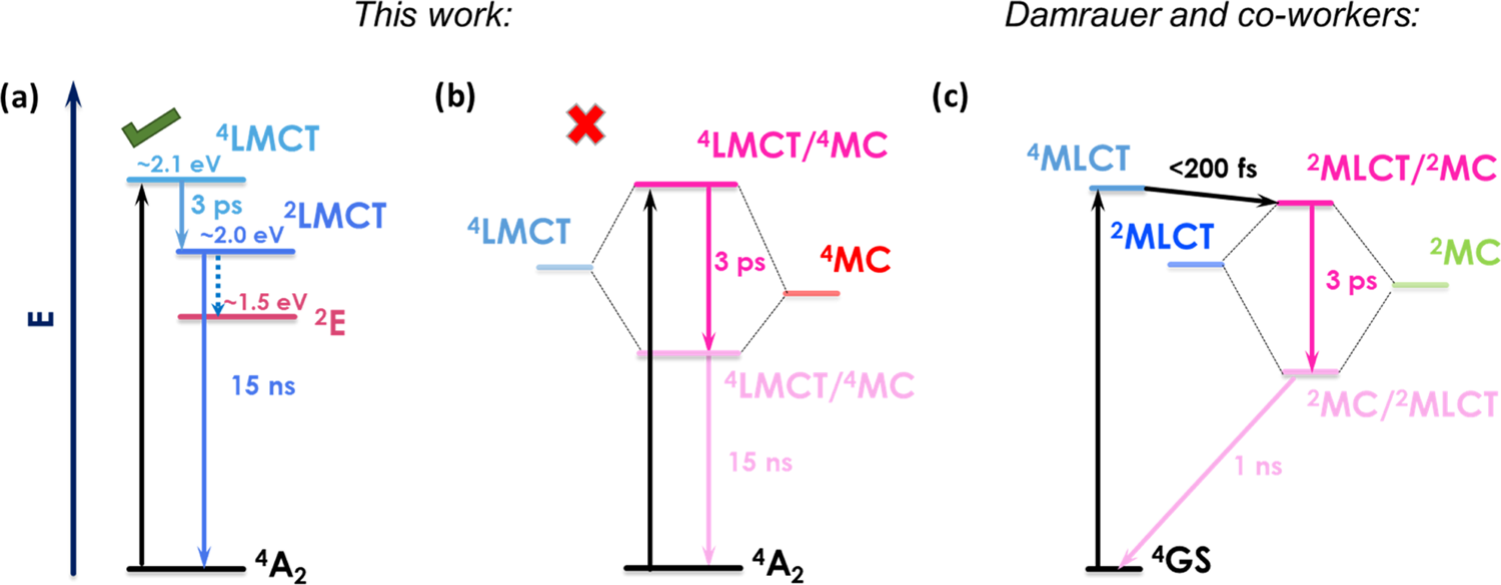
(a) Jablonski diagram depicting the various photophysical transitions observed for [Mn^IV^L_2_]^2+^. Note that state labels can be expected to not be strictly applicable due to mixing. (b) Alternative state mixing hypothesis to account for the observations was ruled out as a possibility in this complex; see the main text for details. (c) Photophysical picture established for related d^3^ V(II) complexes (adapted from ref (31)) for a ready comparison with (a, b).

Additional remarks must be made to complete the commentary on the peculiar photophysics observed in this complex. Given the propensity of this ligand-set to destabilize MC states, a natural question emerges: is state mixing with the ^4^LMCT a possibility? This would present a photophysical landscape (Figure 4b) similar to that observed for aforementioned V(II) complexes^31^ (Figure 4c) but with state mixing of the quartet ^4^LMCT/^4^MC states instead of the doublet ones, and the 3 ps component characterizing conversion from the upper to lower ^4^LMCT/^4^MC. Two points can be thought to speak against this hypothesis: first, it seems less likely that the lower ^4^LMCT/^4^MC state should be a long-lived dark state, in light of the fact that the upper state exhibits at least some extent of radiative coupling to the ground state. Second, there is an overall lower precedence and likelihood of long-lived CT states sharing the ground state’s multiplicity. Finally, unlike the entirely dark V(II) complexes, stimulated emission is clearly observed here on initial time scales. This serves as critical evidence of the initially populated state being a quartet instead of a doublet, and the scenario depicted in Figure 4c may be safely precluded.

On the other hand, magnetic circular dichroism measurements previously conducted on the complex^22^ exhibit sharp lineshapes on the lower-energy side of the LMCT band, making the involvement of spin-forbidden metal-centered states plausible. Excitation at 550 nm into the lower-energy shoulder indeed revealed a reduction in signal amplitude of the initially formed CT state, although spectral signatures and kinetic parameters remained identical (Figure S9). Thus, the CT state having some metal-centered character cannot be ruled out.

An attempt was made to measure a drop-cast film sample to observe spectral signatures exclusive to the ^2^E state but proved challenging due to sample degradation. Nevertheless, the data are presented in Figure S7, and at least confirm the same initial 3 ps component for the assigned intersystem crossing. Interestingly, on longer time scales (>1 ns), no drastic spectral differences are seen, apart from the bleach naturally expected from the broadened charge-transfer absorption observed in the film. Given the reactivity (see below), a predominantly ^2^LMCT assignment still remains the most reasonable option with the collected data set.

### Reactivity

**Figure 5:**
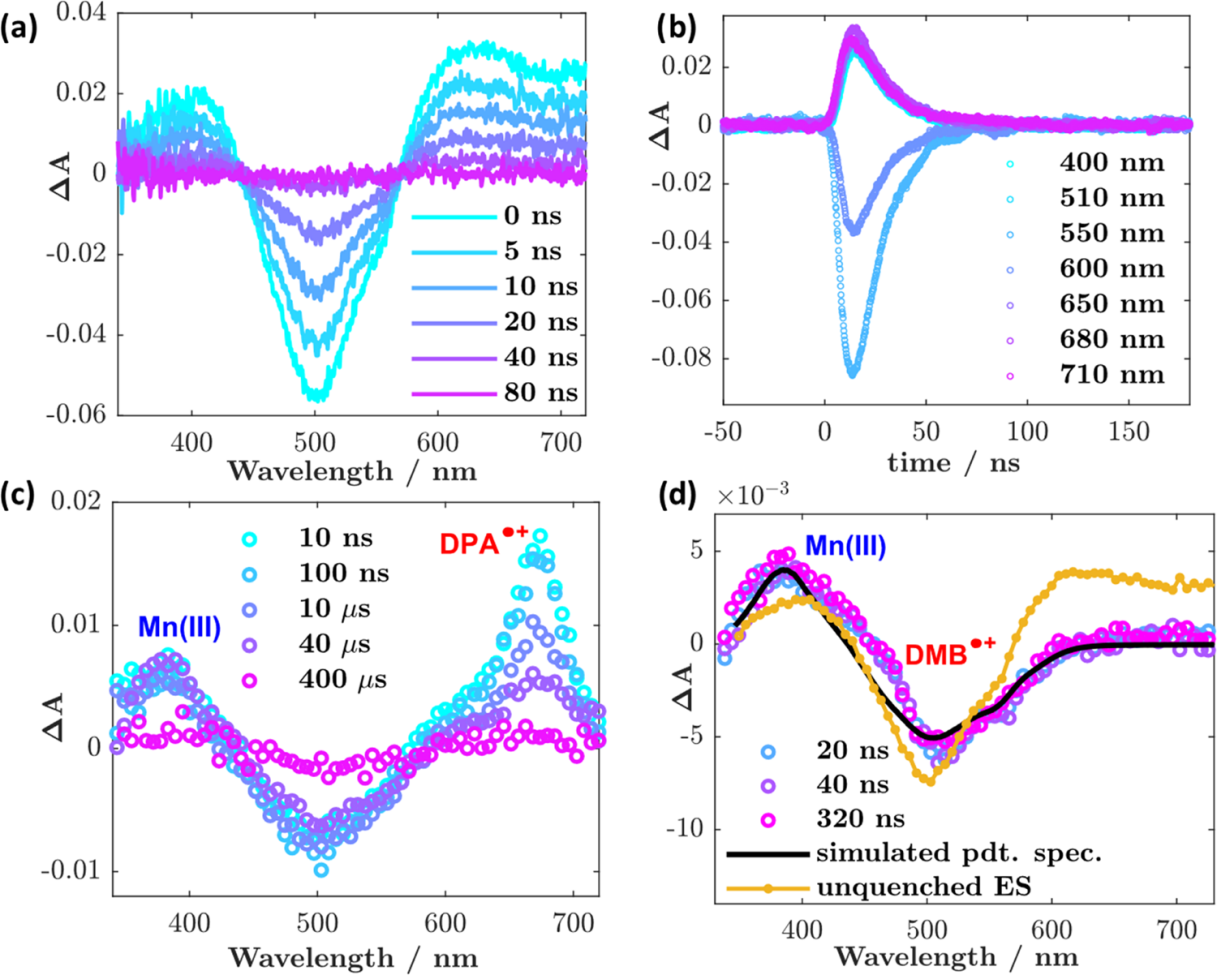
(a) Nanosecond transient absorption spectra at selected time points recorded for [Mn^IV^L_2_]^2+^ in acetonitrile (excitation wavelength = 480 nm, pulse energy = 12 mJ/pulse, absorption ≈0.4 at the excitation wavelength, and integration time = 10 ns). (b) Kinetics monitored at various wavelengths (spectral bandwidth = 5 nm). (c, d) Spectral data for reductive electron transfer quenching of *[Mn^IV^L_2_]^2+^ by diphenylamine (DPA) and 1,3–dimethoxybenzene (DMB), respectively. DPA conc. = 100 mM, DMB = 400 mM. Excitation wavelength = 480 nm, pulse energy = 10–12 mJ/pulse, absorption ≈0.3–0.4 at the excitation wavelength, and integration time = 10–40 ns.

The excited state is readily reductively quenched by diphenylamine (Figure 5c; +0.45 V (0.83 V vs SCE)^57^), confirmed by the observed transient signals for the reduced complex at 380 nm and the diphenylamine radical cation (DPA^+^) at 685 nm; details of the recombination kinetics can be seen in Figure S14. Using available differential extinction coefficients for the products and [Ru(bpy)_3_]^2+^ as a relative actinometer, the total yield can be determined to ∼3% (Figure S14). Inasmuch as the encounter complex is a doublet (*S* = 1/2), recombination to the quartet ground state (*S* = 3/2) should be spin-forbidden. Despite this fact, the product yield is very similar to that obtained for the iron analogue,^23,58^ where the recombination should be spin-allowed. Alternative recombination pathways may be considered to resolve the conundrum, e.g., to the ^2^T or ^2^E state: the latter can be estimated to lie 1.5 eV above the ground state based on the emission data (Figure 1b). The driving force for the charge separation process, Δ*G*_CS_, when DPA is the quencher is approximately −1.0 eV. The driving force for recombination is therefore around −1.1 eV, and the population of the aforementioned states would appear to be too uphill to be relevant.

On the other hand, *[Mn^IV^L_2_]^2+^ can also successfully oxidize other organic substrates, such as 1,3-dimethoxybenzene (Figure 5d) and 4-methylanisole (Figure S15), with potentials of 1.22 and 1.42 (1.6 and 1.8 V vs SCE), respectively.^59^ Solvents DMSO and DMF can also be oxidized, where the characteristic absorption of [Mn^III^L_2_]^+^ is evident (Figure S17). This demonstrates the practical applicability of this complex to accomplish energy-demanding photooxidation reactions. This reactivity data and yield also serve to provide indirect proof of a substantial proportion of the excited state being long-lived. Indeed, the observed changes in the transient signals occur on time scales which imply diffusional electron transfer reactivity and cannot be attributed to purely static quenching of the ^4^LMCT.

Interestingly, the ^2^T and ^2^E states themselves may possess considerable potential for reactivity. Wenger and co-workers have reported a potential of 0.88 for the ^2^E state of [Cr(dqp)_2_]^3+^, finding that the Weller formalism may be applied to good accuracy also for MC states.^60^ Accordingly, a rather high reduction potential in the vicinity of 1.12 V may be estimated also for the ^2^E state of [Mn^IV^L_2_]^2+^. Thus, in the less likely scenario that the 15 ns state is ligand field in character, one may still expect remarkable photosensitization ability, although the observed oxidation of 1,3-dimethoxybenzene and 4-methylanisole should not be in reach of the ^2^E state. As for the ^2^LMCT excited state assignment, rather strongly indicated by the cumulative data set, potentials in excess of 1.12 V but less than or close to 1.42 V are accessible for practical use. This also provisionally positions the ^2^LMCT above the ^2^E state energetically, an observation supported by the computed one-dimensional (1D) potential energy surfaces for the various electronic states accessible in the complex (Figure S25). The thermal population of the ^2^E via the ^2^LMCT may not be precluded, and it may serve as a potential nonradiative decay pathway, in any case.

## Conclusions and Outlook

To summarize, the solution phase photophysics of a Mn(IV)-carbene complex has been elucidated, and its excited state reactivity confirmed, making it one of only two Mn-based photosensitizers known so far.^7^ The photophysical landscape is atypical, where the highly energetic long-lived ^2^LMCT state is dark. At the same time, the doublet-to-quartet spin-forbidden transition to the ground state presents an elegant parallel of the triplet-to-singlet transition often seen in the charge-transfer excited states of Ir- or Ru-based sensitizers. This is in contrast to the properties of similar Cr-based d^3^ complexes,^53,61^ which are prone to exhibit spin-flip emissions based exclusively on the metal center. [Mn^IV^L_2_]^2+^ does not preclude even this possibility, of course, with its first report presenting a detailed characterization of the near-infrared ^2^E emission in the solid state.^22^

Apart from emission quantum yield, the Mn(IV)-carbene presents sizable improvements in several properties compared to its Fe(III) and Co(III) analogues: an excited state which is a highly potent photooxidant, with over twice the absorptivity for the CT transition, together with an enhanced lifetime of 15 ns for its longest-lived predominantly CT excited state. It also retains the remarkable photostability in solution known for this subset of complexes. In terms of prospects for application, it is also worthwhile to mention that the properties of the other two potentially accessible oxidation states (Mn(III) and Mn(II)) of this complex are also promising, with prominent absorptivities and couple potentials, which indicate competent excited states. It would be remiss to not mention that, based on the *E*^0^ and *E*_0–0_ values, Mn(III)-carbene can also be expected to undergo symmetry-breaking charge separation (*Mn^III^ + Mn^III^ → Mn^IV^ + Mn^II^) much as Fe(III)-carbene^62^—a possibility we hope to examine in future work.

In closing, a few reflective remarks are in order. For the longest time, ligand-field states have been the bane of first-row transition metal photophysics: much too low in energy to be photochemically useful themselves and providing highly efficient decay pathways for energetic charge-transfer states. The findings presented here do not challenge this view per se, but certainly present an opportunity for re-examination: the reactivity of the quintet ligand-field state of [Fe(tren(py)_3_)]^2+^ has been reported,^63^ for instance. Even in the present study, both the mixing and potential coupling of MC states with LMCT states cannot be ruled out. From this vantage point, ligand-field states also deserve a closer look. It must be acknowledged, however, that ligand-field transitions have extremely poor molar absorptivity (10–100 M^–1^ cm^–1^). Particularly in cases such as recently advanced conventional Co-polypyridyls,^64^ this deficiency cannot be overcome without attaching an additional light absorber and generating an assembly. By contrast, the present study highlights that the trifecta of high visible molar absorptivity, long lifetime, and high excited state energies—those of CT or ligand-field states—can be collectively realized in a robust mononuclear motif, based on an earth-abundant metal. The key is judicious ligand design. In that regard, the *tris*(imidazol-2-ylidene)borate ligand is in a class of its own when it comes to steric integrity, ligand-field strength, and possibilities for fine-tuning: ligation with different metals has given rise to remarkable photophysics so far, and it would appear only more is to follow.

## Materials and Methods

### Chemicals

All materials were used as-received without further purification, and spectroscopic grade solvents were purchased from Sigma-Aldrich whenever available: this included acetonitrile, methanol, dichloromethane, dimethylformamide, and dimethyl sulfoxide. For making 1:4 methanol/ethanol solutions, absolute ethanol was used. The title compound, [Mn^IV^L_2_](PF_6_)_2_, was synthesized according to literature procedures (ref (21)).

### Steady State Absorption and Emission Spectroscopy

Absorption measurements were carried out on Varian Cary 50 and 5000 spectrophotometers in 1 cm × 1 cm quartz cuvettes in the solution phase, and for the solid state, the film formed by drop-casting on a cover glass slide was placed in the analyzing beam path using a solid state sample holder. Emission and excitation measurements were undertaken on a Horiba Jobin Yvon Fluorolog using a standard right-angle geometry for solution phase measurements and front face detector geometry (60° angle) for film measurements. The signals were corrected for fluctuations in the light source and detector response. Excitation and emission slit widths corresponding to spectral resolutions of 5 and 8 nm, respectively, were used, and the integration time was 1 s.

### Transient Absorption Spectroscopy

Transient absorption measurements on femtosecond and nanosecond time scales were carried out on setups previously described.^62^ Briefly: the output of a Coherent Libra amplifier (3 kHz, ca. 45 fs pulses) with an integrated oscillator and pump lasers was split into a pump and probe beam. The excitation wavelengths in the visible were generated by directing the pump beam into the optical parametric amplifiers (TOPAS-C, Light Conversion), while the fundamental of the amplifier was focused onto a circular CaF_2_ plate in order to generate the white light supercontinuum in the ranges of 340 to 740 nm. The probe spectrum was detected by using a custom-made silicon diode array from Newport. Pump–probe overlap was optimized for the sample, and the pump power was adjusted to 1 mW. A mechanical optical delay stage was used to collect data at different time points by varying the delay of the probe with respect to the pump, and a range of −5 ps to 8 ns was scanned. Sequential mode was used for solution and random sampling for film. Five scans were averaged for the solution phase measurements; for the film, owing to degradation, multiple measurements were carried out at different time-point densities, and every new scan was made on a fresh spot.

A Q-switched Nd:YAG laser (Model NT342B, EKSPLA; fwhm = 8 ns) was used as the pump source for the nanosecond measurements. The fundamental output at 1064 nm was frequency tripled and redirected to pump an optical parametric oscillator (OPO) equipped with type II nonlinear BBO crystals to produce the desired pump wavelength in the visible range for sample excitation. The repetition rate was 10 Hz, and data acquisition was carried out at 1 Hz with the help of electronically controlled shutters. The pump energy was ca. 12 mJ/pulse (±20%) for the solution phase measurements (made in right-angle detection geometry) and at ca. 4 mJ/pulse for the film measurements (made with a 60° angle between pump and probe). The probe light from the Xe arc lamp was pulsed except for long time scale measurements (>400 μs) where a continuous wave probe was used.

The LP920 detection system (Edinburgh Instruments) equipped with a photomultiplier tube and an Andor iStar CCD camera (cooled to −8 °C) was used to acquire kinetic and spectral data, which was collected using the L900 software (which controlled the laser, shutters, Tektronix digital oscilloscope, and the CCD camera) on the connected computer. The kinetic traces at a given wavelength were recorded with a consistent resolution of 5 nm, and for spectral measurements, the monochromator was positioned at a center wavelength of 575 nm. Measurements were typically averaged for 16 shots.

### Electrochemistry and Spectroelectrochemistry

Electrochemical measurements were carried out in acetonitrile with 0.1 M TBAPF_6_ (tetrabutylammonium hexafluorophosphate) as supporting electrolyte, with a custom-made quartz cell utilizing a standard three-electrode setup consisting of a Ag/Ag^+^ reference electrode (0.01 M AgNO_3_ in acetonitrile), platinum counter electrode, and glassy carbon working electrode (1 mm, CH Instruments). Spectroscopic grade acetonitrile was dried for 48 h over 3 Å activated molecular sieves, and electrochemical grade TBAPF_6_ (Sigma) was dried for 24 h under vacuum at 80 °C prior to use. The working electrode was polished by using alumina paste between each measurement. Solvent-saturated argon was used to purge the sample, and a blanket thereof was maintained in the cell headspace. Cyclic voltammograms were recorded at a scan rate of 100 mV/s, and the differential pulse voltammogram parameters were as follows; step potential: 5 mV, modulation amplitude: 25 mV, modulation time: 0.05 s, interval time: 0.1 s. UV–vis spectroelectrochemistry was carried out in the same cell with the same reference electrode, but a platinum mesh electrode inserted in the 1 mm optical path served as a working electrode instead to perform bulk electrolysis. An Autolab potentiostat (PGSTAT302) was used to control the three-electrode setup using GPES 4.9 software, and an Agilent 8453 diode array spectrophotometer was used to record the spectral traces.

### Electronic Structure Calculations

Mn complexes and Zn complexes were optimized employing B3LYP functional^65−68^ with Grimme’s D3 dispersion correction.^69^ The SDD effective core potential (ECP) and associated basis sets were used for Mn and Zn atoms,^70^ while the 6-311G* basis set was utilized for all other atoms (Cl, B, N, C, and H).^71^ Solvent effects (acetonitrile) were included in the calculations through polarizable continuum model (PCM).^72^ The ultrafine grid was used for the calculations. Vibrational frequency analysis was performed on optimized structures to confirm their convergence to the local minima at their respective potential energy surfaces. Natural orbital (NO) analysis was applied to determine the character of the open-shell electronic states. Mulliken population analysis implemented in the AOMix program^73,74^ was employed to determine the localization of molecular orbitals on molecular fragments in the quartet ground state (Mn) and singlet ground state (Zn).

Time-dependent density functional theory (TD-DFT)^75^ was employed for excited state analysis, using the same level of theory as the ground state calculations. The stick spectrum was broadened by using Lorentzian functions with a half-width at half-maximum (HWHM) of 0.12 eV. Solvent effects (acetonitrile) were included via the polarizable continuum model (PCM). Natural transition orbitals^76^ (NTOs) were used to characterize the transitions in each excited state for Zn complexes. All calculations were carried out using the Gaussian 16 software package (Revision A.03).^77^

## Supporting Information Available

Experimental details, additional steady state and time-resolved spectroscopic data, actinometry, fitting procedures, and reactivity with other substrates (PDF)
Structures of the complexes mentioned in this work (XYZ)

## Supplementary Material

ja4c08588_si_001.pdf

ja4c08588_si_002.xyz
