# Supplementary material for: Enter MnIV–NHC: A Dark Photooxidant with a Long-Lived Charge-Transfer Excited State
Source: J Am Chem Soc. 2024 Aug 6;146(35):24619–29. doi: 10.1021/jacs.4c08588 (PMC11378296; doi:10.1021/jacs.4c08588)
Supplement: Supplementary file 1 — ja4c08588_si_001.pdf [file ja4c08588_si_001.pdf]

Supporting Information:

Enter MnIV-NHC: A Dark Photo-oxidant with a Long-Lived Charge Transfer Excited State

Nidhi Kaul[1], Eyram Asempa[2], Juan A. Valdez-Moreira[3], Jeremy M. Smith[3], Elena Jakubikova[2], Leif Hammarström[1]*

[1]Department of Chemistry – Ångström Laboratory, Uppsala University, Box 523, SE-75120 Uppsala, Sweden

[2]Department of Chemistry, North Carolina State University, Raleigh, North Carolina 27695, USA

[3]Department of Chemistry, Indiana University, 800 East Kirkwood Avenue, Bloomington, Indiana 47405, USA

*Corresponding Author:

Leif Hammarström (leif.hammarstrom@kemi.uu.se)

Contents

**1.**	**Steady-State Spectroscopy**	S3
**2.**	**Electrochemistry and Spectroelectrochemistry**	S4
**3.**	**Transient Absorption Spectroscopy**	S5
**4.**	**Reactivity**	S9
**5.**	**Time-Dependent Density Functional Theory**	S14
**6.**	**References**	S17

1. Steady-State Spectroscopy

1.1. Solid-state

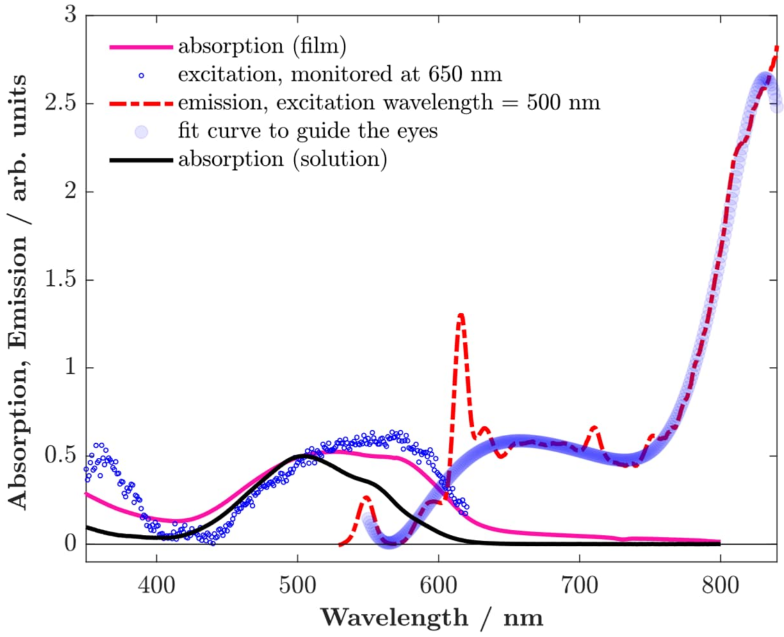

Figure S1. Absorption (pink), emission (dashed red, with blue fit line to guide the eyes), and excitation (blue dots) data for $[Mn^{IV}L_2](PF_6)_2$ for a solid state sample (dropcast film on glass substrate). Absorption in acetonitrile presented in black for comparison.

1.2. Solution

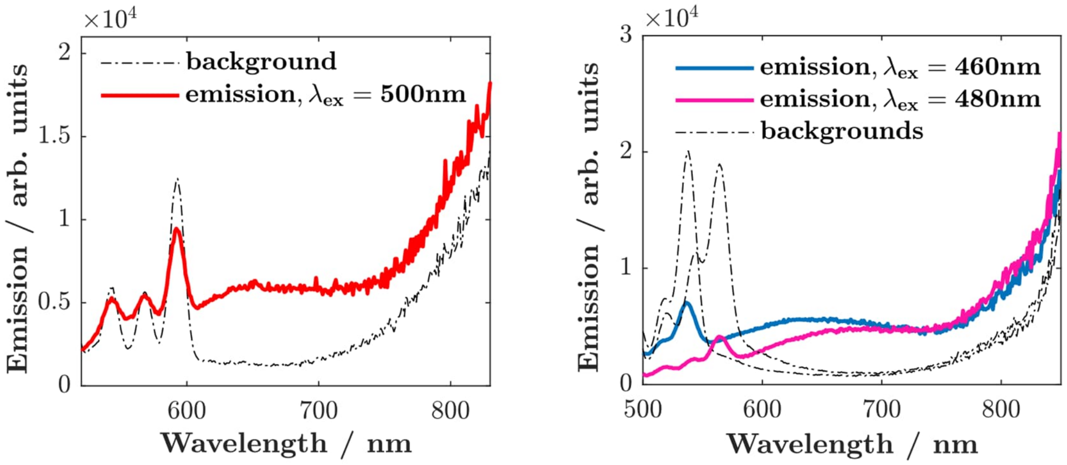

Figure S2. Emission spectra of $[Mn^{IV}L_2]^{2+}$ in a ca. 80 µM solution in acetonitrile (solid red), plotted together with solvent background (both corrected for detector response, resulting in the rise seen towards the red). *Left:* λ_{ex} = 500 nm, excitation and emission slit widths corresponding to a spectral resolution of 5 and 8 nm, respectively. *Right:* λ_{ex} = 460 and 480 nm (blue and pink), excitation and emission slit widths corresponding to a spectral resolution of 8 nm.

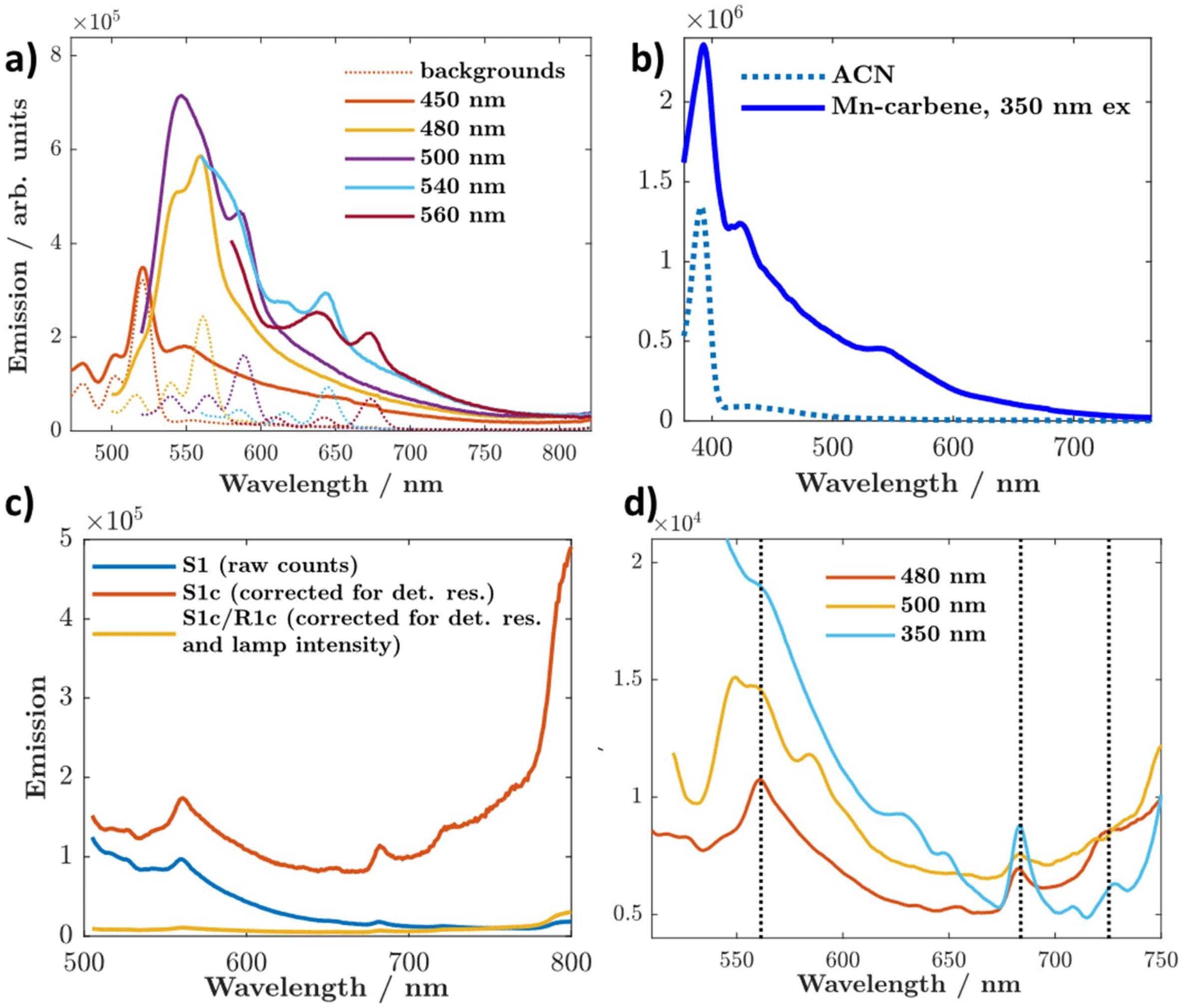

Figure S3. *a) and b):* Emission spectra of $[Mn^{IV}L_2]^{2+}$ in a ca. 20 µM solution in acetonitrile, at indicated excitation wavelengths in the legend, plotted together with solvent background. Excitation and emission slit widths of 5 and 8 nm, respectively. Note the variation in band maximum due to contributions from the Raman scatter. *c):* Emission spectra of $[Mn^{IV}L_2]^{2+}$ in a ca. 20 µM solution in 4:1 Ethanol:Methanol glass, recorded at 77K. Excitation and emission slit-widths of 5 and 8 nm, respectively. Excitation wavelength = 480 nm. Raw counts clearly show the NIR emission at low temperature. *d):* Emission spectra of $[Mn^{IV}L_2]^{2+}$ in a ca. 20 µM solution in 4:1 Ethanol:Methanol glass, recorded at 77K. Excitation and emission slit-widths of 5 and 8 nm, respectively. Excitation wavelengths indicated in the legend. Dotted black vertical lines indicated structural features in the emission independent of the excitation wavelength.

Using the known quantum yield of 2.1% the iron analogue[1], $[Fe^{III}L_2]^{+}$, an estimate could be made for the quantum yield of $[Mn^{IV}L_2]^{2+}$. The relevant data is shown in Figure S4. Due to the orders of magnitude higher emission of the iron complex (and that of other commonly used reference standard fluorophores), a solution was instead used where it was quenched down to 11.25% of its emission value by Indole. The data nevertheless needs to be scaled by a factor of 0.1 to be on the same scale (Figure S4, left) when using the same excitation slit-widths and integration time of 1 s. The emission slit-widths were 5 and 8 nm for iron and manganese complex, respectively. All other conditions remaining same, the quantum yield can therefore be calculated using:

$$\phi_x = \phi_s \times \frac{I_x}{I_s} \times 0.1125 \times 0.1 \times 0.39$$

where a quadratic correlation between intensity and slit-width is assumed to yield the factor of 0.39 for the difference in emission slit-widths, and 0.1125 and 0.1 account for the quenching and scaling, respectively. ϕ_s=0.021, and the integrated emission intensities are I_s = 4.11 × 10^7 and I_x = 3.85 × 10^7 for the iron and manganese complex, respectively. The determined quantum yield is thus ca. 9.8 × 10^{-5}, and the estimated lifetime from the Strickler-Berg relation is 4.1 picoseconds (see main text).

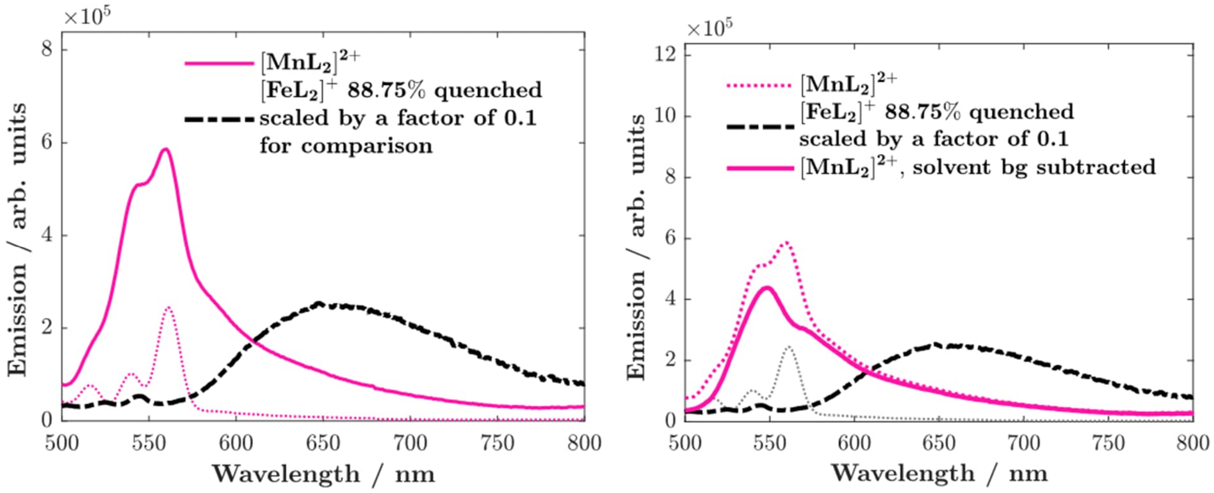

Figure S4. *Left and right:* Comparison of emission spectra of $[Mn^{IV}L_2]^{2+}$ and $[Fe^{III}L_2]^{+}$ in acetonitrile, absorption = 0.1 at the excitation wavelength. Excitation slit-width = 5 nm, and emission slit-width = 5 nm and 8 nm for $[Fe^{III}L_2]^{+}$ and $[Mn^{IV}L_2]^{2+}$, respectively. See associated text for details.

2. Electrochemistry and Spectroelectrochemistry

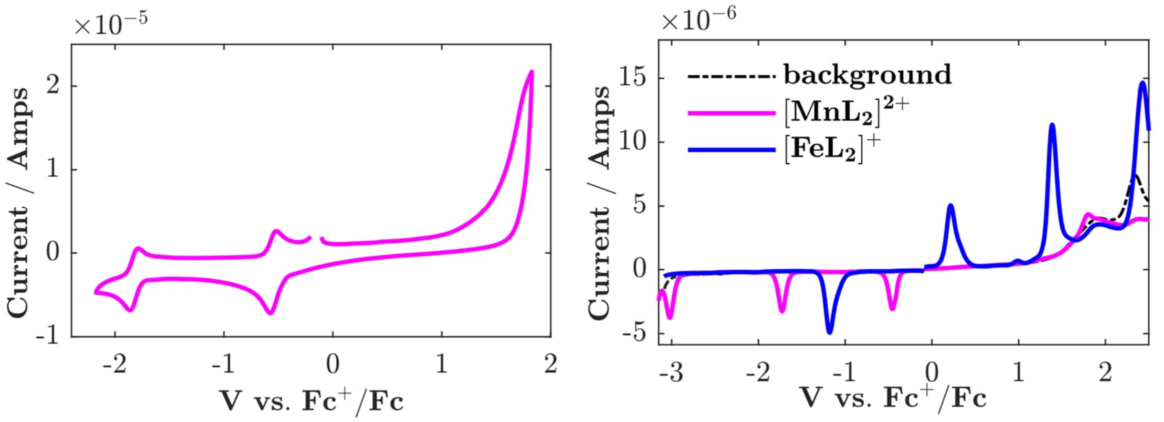

Figure S5. *Left:* Cyclic voltammetry (100 mV/s) and, *Right:* differential pulse voltammetry data for ~1 mM $[Mn^{IV}L_2]^{2+}$ (pink) plotted together with $[Fe^{III}L_2]^{+}$ (blue), to show the ligand oxidation. Solvent: acetonitrile, with 0.1 M $TBAPF_6$ as supporting electrolyte.

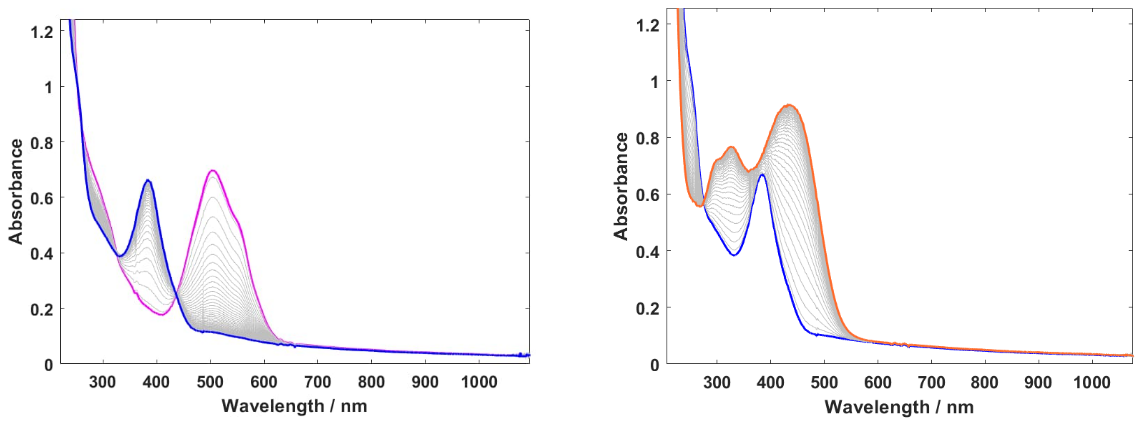

Figure S6. Spectral signatures of $[Mn^{III}L_2]^{+}$ (left, blue) and $[Mn^{II}L_2]^{0}$ (right, orange), as determined from controlled potential electrolysis of a ca. 1 mM solution of $[Mn^{IV}L_2]^{2+}$ in acetonitrile with 0.1 M $TBAPF_6$ as supporting electrolyte, at –0.8 V and –2 V, respectively.

3. Transient Absorption Spectroscopy

3.1. Solid-state

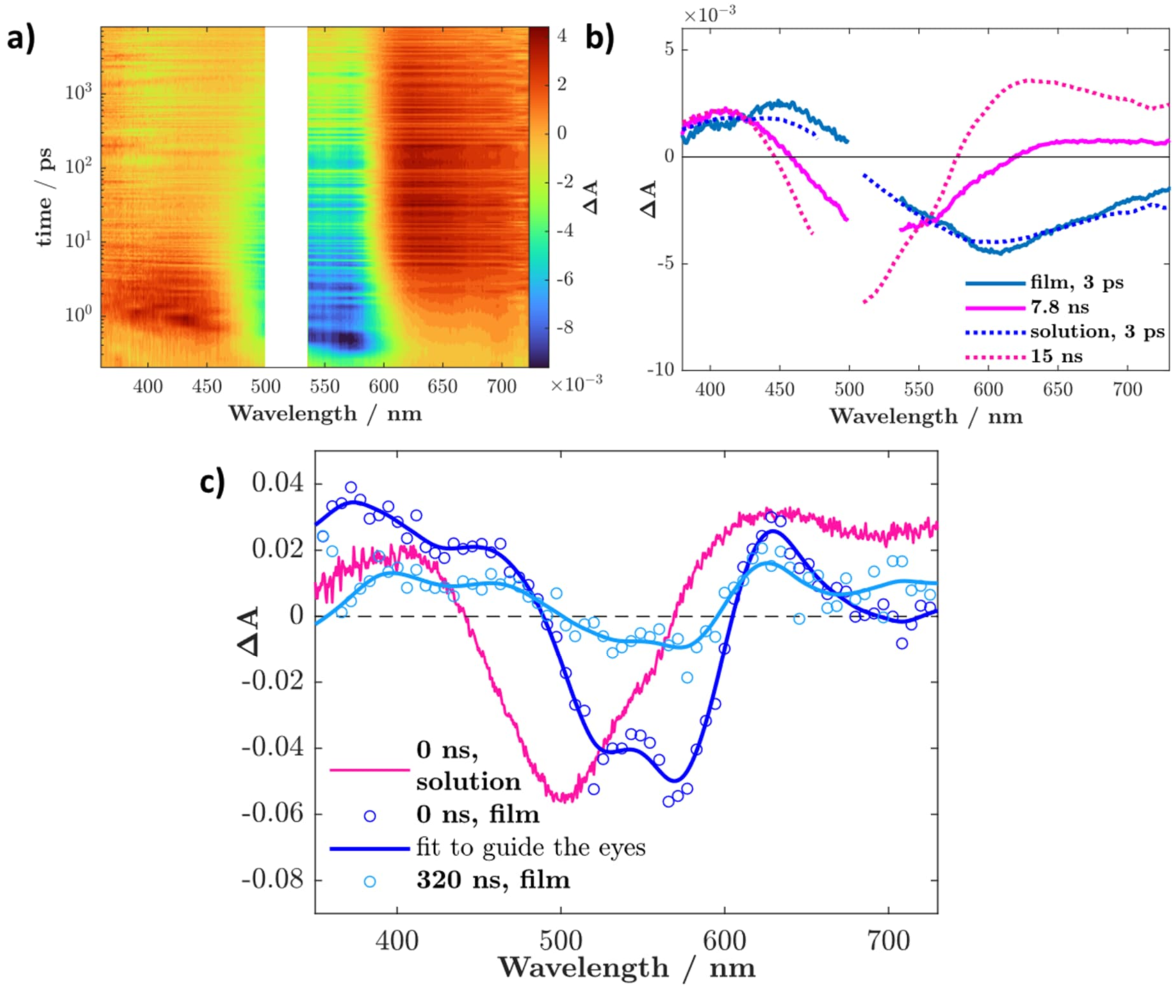

Figure S7. a) Contour map of femtosecond transient absorption data recorded for a $[Mn^{IV}L_2](PF_6)_2$ film dropcast on glass substrate (excitation wavelength = 520 nm, pulse energy ca. 0.25 µJ/pulse). The data was collected by random sampling instead of a typical sequential measurement to minimize impact from degradation; however, an additional component of ca. 70 ps corresponding to the degradation product could be extracted from the global analysis of the data (not shown) which varied with time-point density and collection time. The decay associated spectra associated with the unchanged, fixed components of ca. 3 ps and 8 ns are presented in panel **b)** (solid blue and pink traces, respectively), together with DAS from the solution phase (dotted, same colours) for comparison. Reasonable agreement can be seen. **c)** Flash photolysis spectral traces at selected time points measured for the solid-state sample (open circles, ten data points binned to one together with fit lines to guide the eyes) and solution phase data (pink). Data normalized for comparison.

3.2. Solution

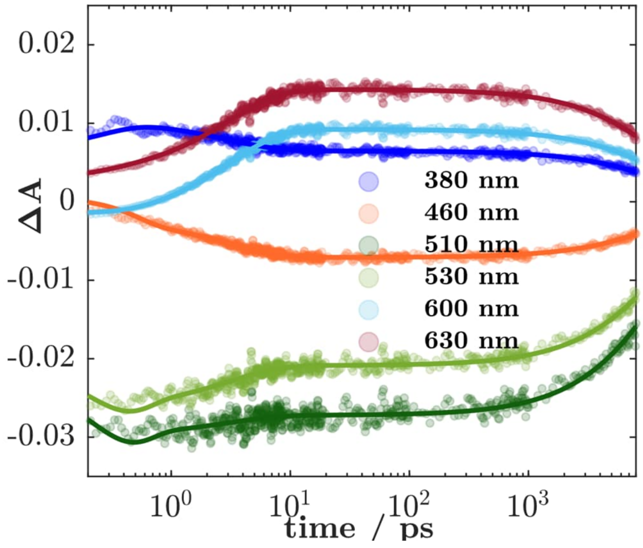

Figure S8. Kinetic traces monitored at indicated wavelengths (vertical cuts from Figure 2a) with solid lines as global fits, resulting in time constants of 3 picoseconds and 15 nanoseconds. Excitation wavelength = 480 nm, pulse energy = 0.7 µJ/pulse, absorption ≈ 0.35 at the excitation wavelength.

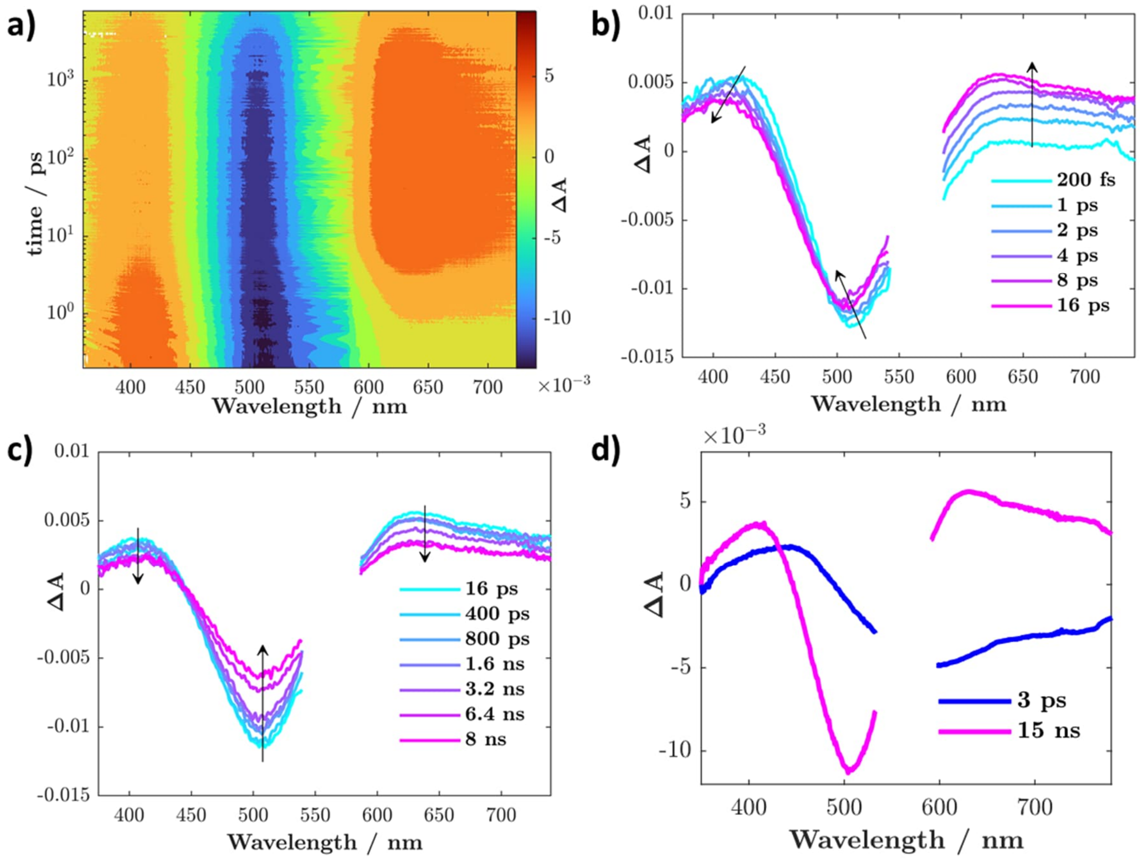

Figure S9. a) Contour map of femtosecond transient absorption data recorded for $[Mn^{IV}L_2]^{2+}$ (excitation wavelength = 550 nm, pulse energy = 0.7 µJ/pulse, absorption ≈ 0.33 at the excitation wavelength). **b)** and **c)** Spectral traces at selected time points (horizontal cuts from a)), with arrows to guide the spectral evolution. **d)** Decay associated spectra obtained from a global analysis of the data in a).

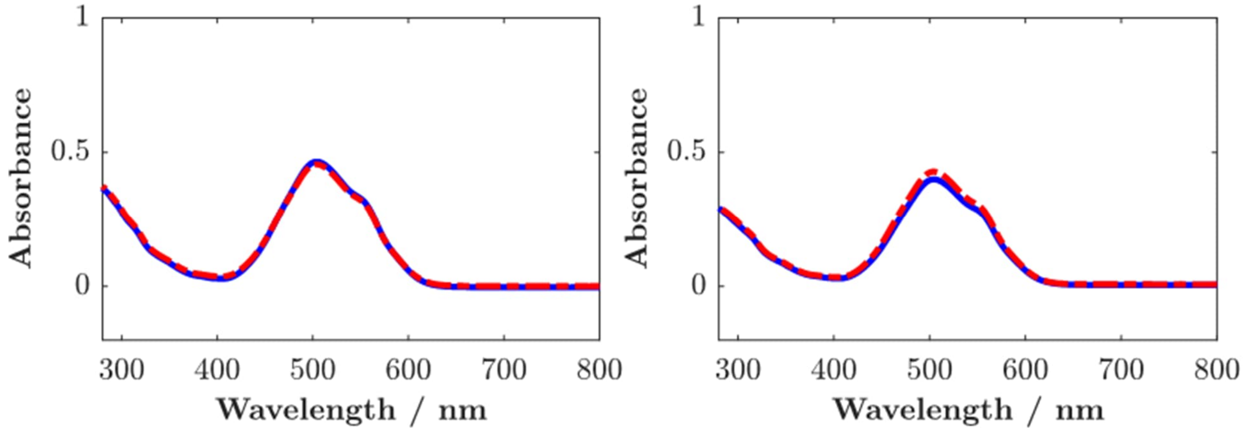

Figure S10. *Left:* Absorption measurement for $[Mn^{IV}L_2]^{2+}$ before (solid blue) and after (dashed red) nanosecond transient absorption measurements (excitation wavelength = 480 nm, pulse energy = 12 mJ/pulse, absorption ≈ 0.4 at excitation wavelength, 1000+ shots). *Right:* Absorption measurement for $[Mn^{IV}L_2]^{2+}$ before (solid blue) and after (dashed red) femtosecond transient absorption measurements (excitation wavelength = 480 nm, pulse energy = 0.7 µJ/pulse, absorption ≈ 0.35 at the excitation wavelength). Note the slight (<0.03) difference in absorption is due to solvent evaporation in the 8+ hour measurement duration.

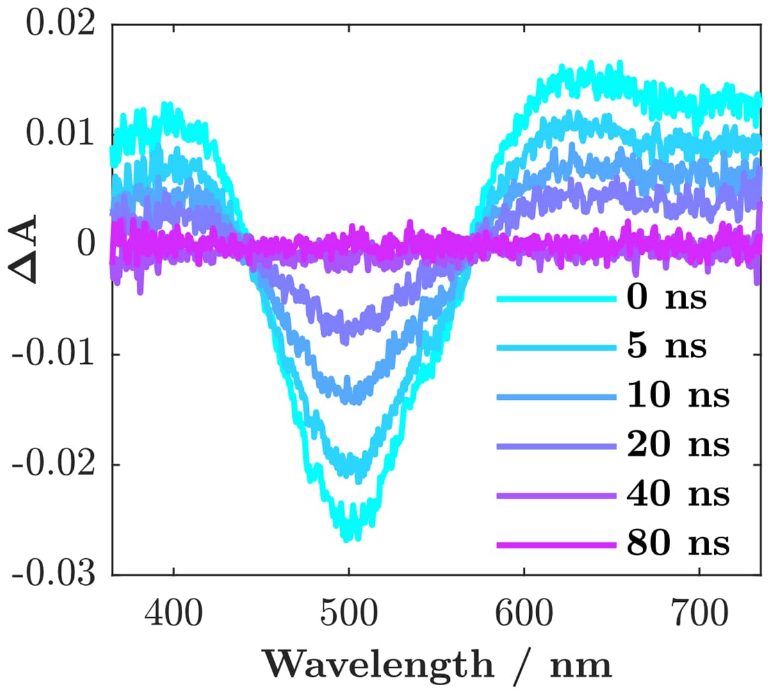

Figure S11. Nanosecond transient absorption spectra at selected time points recorded for $[Mn^{IV}L_2]^{2+}$ in propylene carbonate (excitation wavelength = 480 nm, pulse energy = 10 mJ/pulse, absorption ≈ 0.4 at the excitation wavelength, and integration time = 20 ns)

3.2.1. Fitting

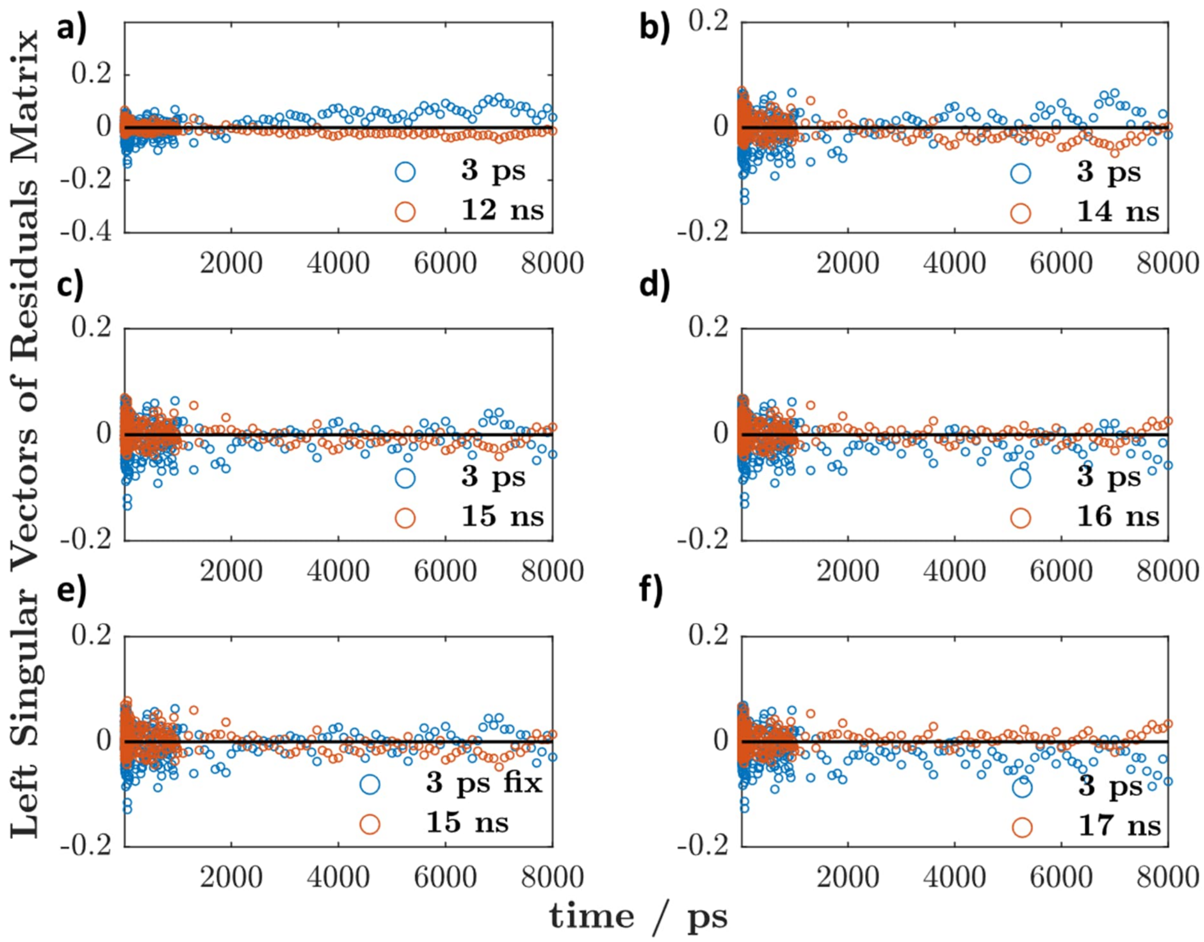

Figure S12. Left singular vectors of the residuals matrix, with time constants indicated in the legend. Apart from panel **e)**, the longer time constant was held fixed.

As noted in the main text, the determined time constant using global analysis is only about half the accessible time window in the fs-TA experiments. Thus, to ascertain the veracity of the value, additional global fits were performed holding the longer time component fixed and leaving free the other fit parameters. The sum of exponentials model employed in Glotaran[2,3] was as previously described (ref 60 and main text). As can be seen in Figure S10, the left singular vectors (LSVs – extracted from the singular value decomposition of the residuals matrix) show systematic deviation about zero for time constants <14 ns and >16 ns. Further, in Figure S10 panel e), the shorter component was held fixed at 3 ps, leading to a convergence at 15 ns, with the LSVs distributed randomly about zero. These observations suggest the global fit convergence to 3 ps and 15 ns is robust.

Further support for the determined time constant could also be found by fitting the ns-TA data, with a representative example shown in Figure S11. In this instance, the FWHM of the laser pulse is ca. 8 ns, so a tail-fit is not adequate. Using convolution with the measured with IRF, time constants of 14 and 16 ns slightly under- and overestimate the decay profile, corroborating a lifetime of 15 ± 1 ns.

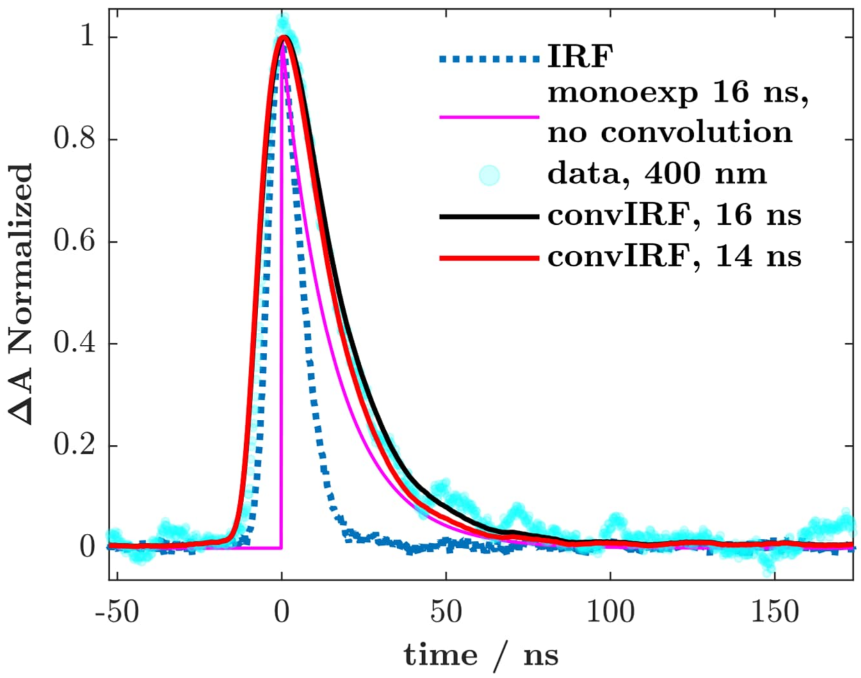

Figure S13. ns-TA kinetics monitored at 400 nm (solid cyan circles) for $[Mn^{IV}L_2]^{2+}$ in acetonitrile (excitation wavelength = 480 nm, pulse energy = 12 mJ/pulse, absorption ≈ 0.4 at the excitation wavelength). IRF (dotted blue). Monoexponential decay with a time constant of 16 ns without convolving with the IRF (solid pink). Monoxponential decays with convolution, time constants 16 ns and 14 ns (solid black and red, respectively).

4. Reactivity

4.1. Actinometry

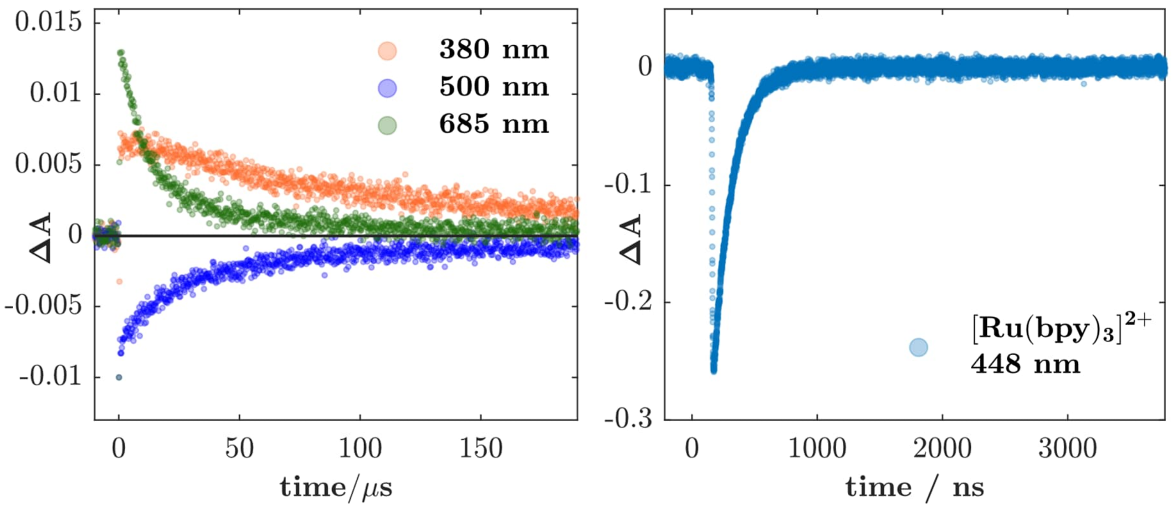

Figure S14. *Left:* The recombination of cage escape products monitored at the indicated wavelengths. The products recombine over a few hundred microseconds, which is broadly compatible with diffusional recombination given the micromolar yields; it can be noted however that the kinetics are incompatible with bimolecular recombination and have different characteristic timescales at different wavelengths. Specifically, the fast decay at 685 nm on a few microsecond timescale, where DPA^+ primarily absorbs, does not represent recombination with its cage escape partner, $[Mn^{III}L_2]^+$; a parent-child reaction and subsequent dimerization is the most likely possibility instead[4], given the quencher excess to ensure complete quenching. *Right:* Kinetic trace for $[Ru(bpy)_3]^{2+}$ at 448 nm recorded in the same conditions as the left panel (with absorption matched at the excitation wavelength), where its differential extinction coefficient is known to good accuracy[5], $\Delta\varepsilon_{448}$= –11300 M^{-1} cm^{-1}. Taken together with $\Delta\varepsilon_{680}$=19200 M^{-1} cm^{-1} for the DPA radical cation[6], a total product yield of 3% can be evaluated.

4.2. Additional Substrates

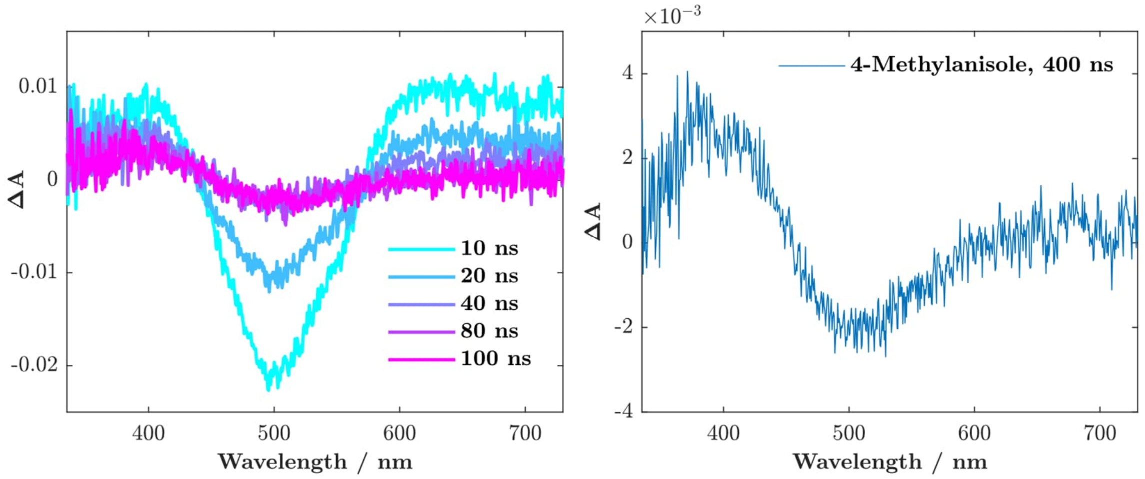

Figure S15. Reductive electron transfer quenching of *$[Mn^{IV}L_2]^{2+}$ by 4–Methylanisole (conc. = 0.8 M) monitored using ns transient absorption spectroscopy. Excitation wavelength = 480 nm, pulse energy = 11 mJ/pulse, absorption ≈ 0.3 – 0.4 at the excitation wavelength, and integration time = 20 ns (left panel) and 100 ns (right panel). Characteristic peaks of $[Mn^{III}L_2]^{+}$ and the 4-Methylanisole radical cation can be seen in the blue.

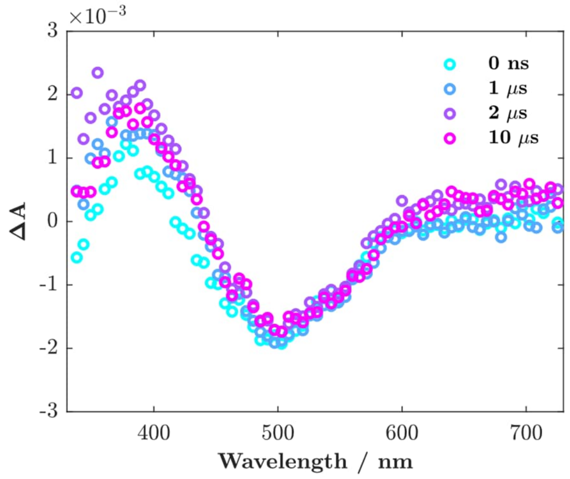

Figure S16. Reductive electron transfer quenching of *$[Mn^{IV}L_2]^{2+}$ by Indole (conc. = 0.2 M) monitored using ns transient absorption spectroscopy. Excitation wavelength = 480 nm, pulse energy = 11 mJ/pulse, absorption ≈ 0.3 – 0.4 at the excitation wavelength, and integration time = 500 ns. The characteristic peak of $[Mn^{III}L_2]^{+}$ can be seen at 380 nm.

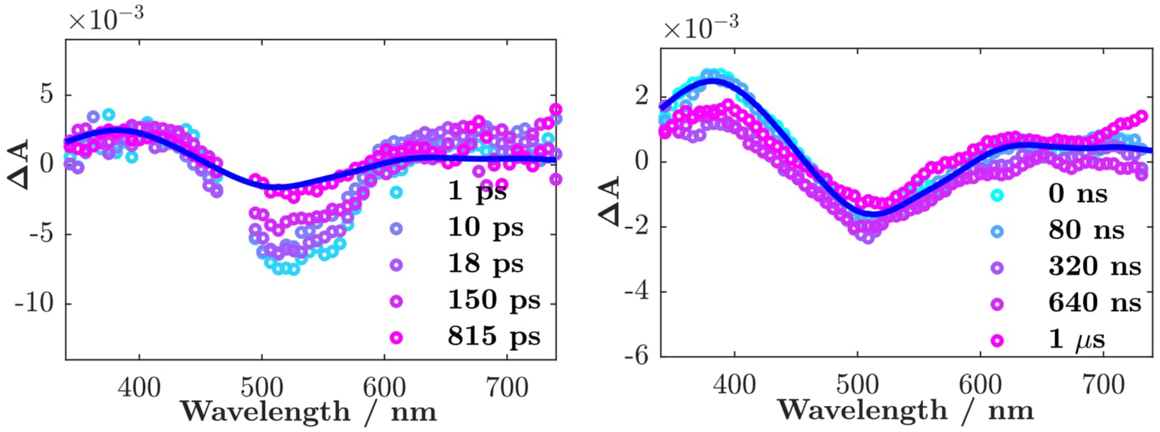

Figure S17. *Left:* Reductive electron transfer quenching of *$[Mn^{IV}L_2]^{2+}$ by DMSO (solvent as quencher) monitored using fs transient absorption spectroscopy. Excitation wavelength = 480 nm, power = 4 mW, absorption = 0.3 – 0.4 at the excitation wavelength. *Right:* Reductive electron transfer quenching of *$[Mn^{IV}L_2]^{2+}$ by DMF (solvent as quencher) monitored using ns transient absorption spectroscopy. Excitation wavelength = 480 nm, power = 10 mJ/pulse, absorption = 0.3 – 0.4 at the excitation wavelength, integration time = 20 ns. Solid blue lines indicate expected product spectrum as determined using spectroelectrochemistry.

Attempts to oxidatively quench *$[Mn^{IV}L_2]^{2+}$, for example using MV^{2+}, failed. These observations, too, are broadly compatible with the results obtained for the iron analogue[7], which is also known to be a relatively poor electron donor. For $[Mn^{IV}L_2]^{2+}$, the quenching product for oxidative quenching would result in a triple positive charge on the complex, which likely detracts from the process altogether.

5. Time-Dependent Density Functional Theory (TD-DFT)

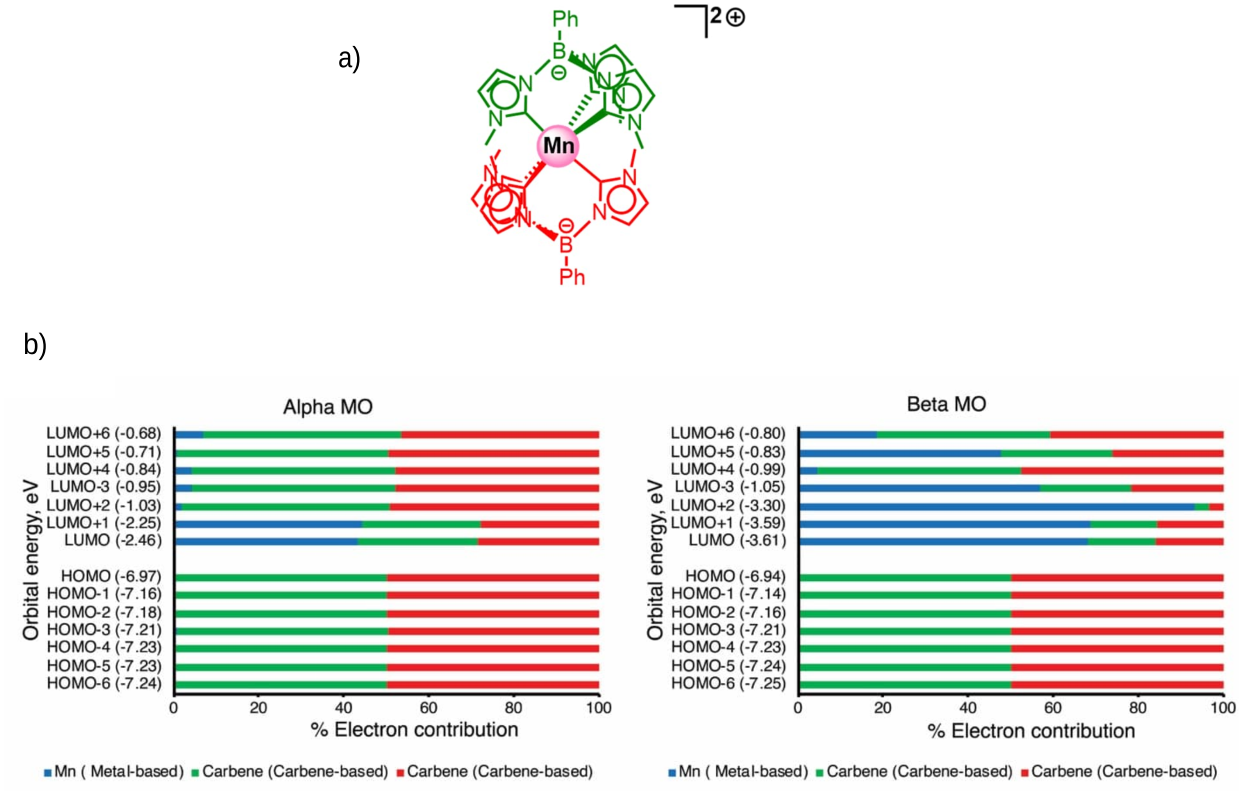

Figure S18. **a)** Fragmentation scheme utilized to determine the localization of MOs in $[Mn^{IV}L_2]^{2+}$. **b)** Fragment orbital analysis of $[Mn^{IV}L_2]^{2+}$. Blue represents the amount of MO electron density on Mn, while green and red denote the amount of electron density on the two carbene-based ligands.

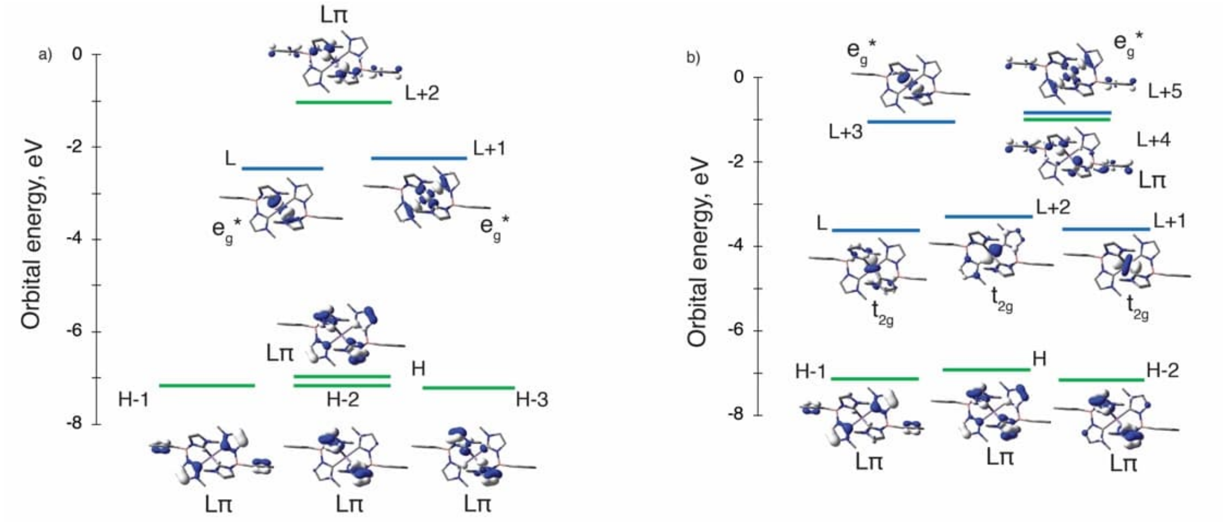

Figure S19. Molecular orbital diagrams of $[Mn^{IV}L_2]^{2+}$. Blue: MOs localized predominantly on Mn, and green: ligand-based MOs.

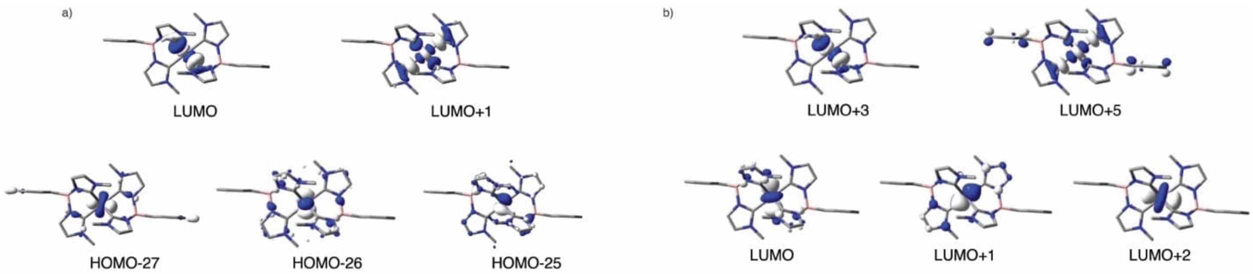

Figure S20. Metal-based t_{2g} and e_g* molecular orbitals of $[Mn^{IV}L_2]^{2\cdot}$ a) alpha and b) beta d-orbitals.

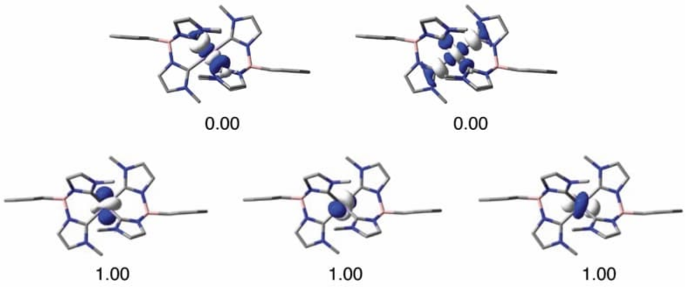

Figure S21. Natural orbital (NO) analysis of quartet state of $[Mn^{IV}L_2]^{2+}$, showing the metal-based natural orbitals. NO analysis suggests that the quartet state has three singly occupied NOs that correspond to d_{xy}, d_{yz} and d_{xz} t_{2g} orbitals of Mn, while the Mn e_g* orbitals ($d_{z}{}^{2}$ and $d_{x}{}^{2}{}_{-y}{}^{2}$) are unoccupied.

Table S1. Hole-particle pairs for the three most intense excited states at band A of $[Mn^{IV}L_2]^{2+}$. (TD-DFT coefficients are shown above the arrows, B represents beta)

Excited State	Hole-particle pair transitions			Character
3	E = 2.70 eV	λ = 459 nm	f = 0.04	
	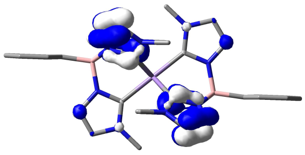<br>HOMO-2B	0.98 →	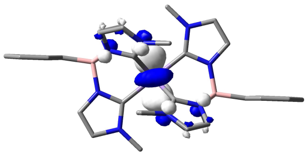<br>LUMO B	96% LMCT
5	E = 2.73 eV	λ = 455 nm	f = 0.12	
	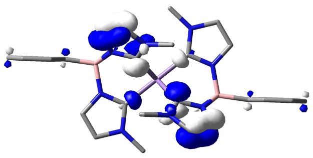<br>HOMO-3B	0.60 →	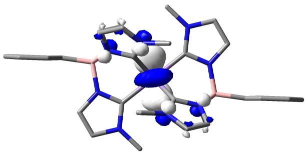<br>LUMO B	36% LMCT
	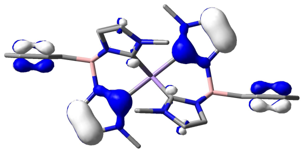<br>HOMO-1B	0.64 →	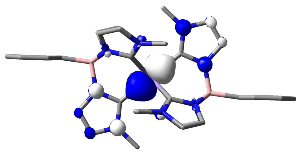<br>LUMO+1B	41% LMCT
6	E = 2.74 eV	λ = 453 nm	f = 0.12	
	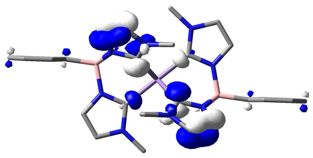<br>HOMO-3B	0.66 →	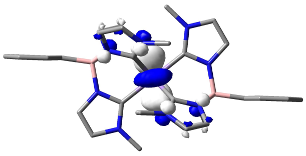<br>LUMO B	43% LMCT
	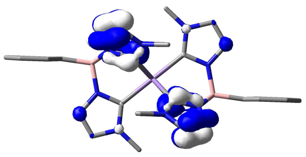<br>HOMO-2B	0.56 →	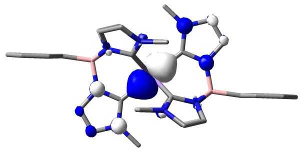<br>LUMO+1B	31% LMCT

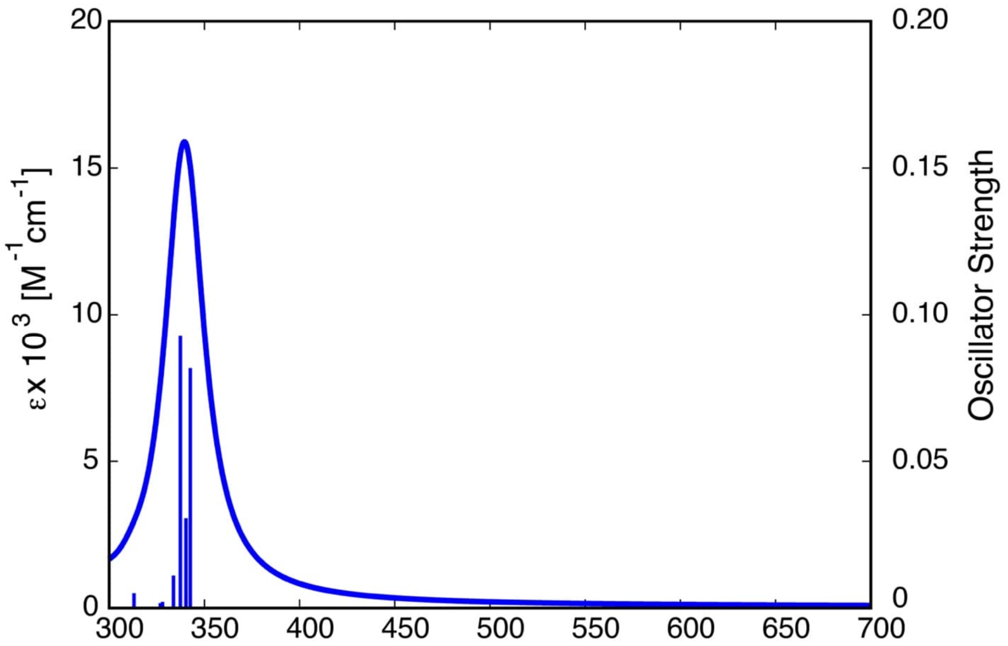

Figure S22. Calculated absorption spectrum with corresponding stick spectrum of $[Mn^{III}L_2]^+$.

Table S2. Hole-particle pairs for the excited state transitions occurring at λ = 343 nm and 337 nm of $[Mn^{III}L_2]^+$. (TD-DFT coefficients are shown above the arrows, B represents beta)

Excited State	Hole-particle pair transitions	Character
9	E = 3.62 eV λ = 343 nm f = 0.08<br>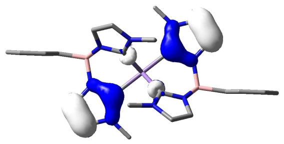 0.93 → 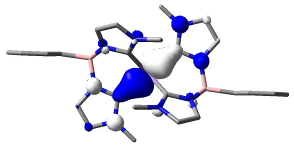<br>HOMO-2B LUMO B	86% LMCT
11	E = 3.67 eV λ = 337 nm f = 0.09<br>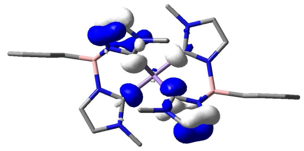 0.87 → 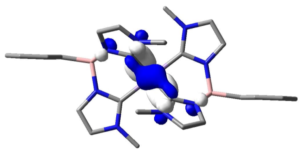<br>HOMO-4B LUMO+1B	75% LMCT

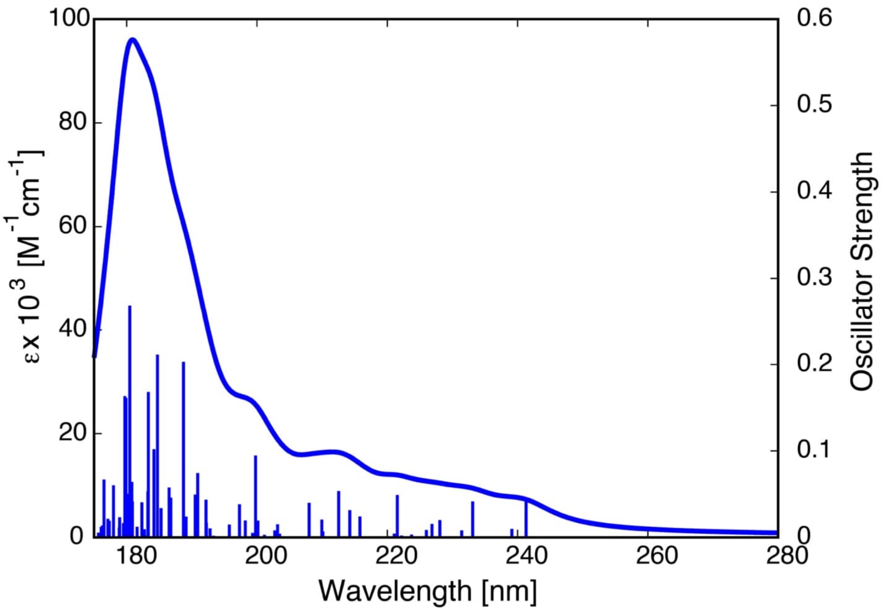

Figure S23. Calculated absorption spectrum with corresponding stick spectrum of $[Zn^{II}(L)Cl]^0$.

Table S3. Natural transition orbital (NTO) analysis for the excited state transitions occurring at λ = 241 nm and 180 nm of $[Zn^{II}(L)Cl]^0$. (NTO coefficients are shown above the arrows)

Excited State	Hole-particle pair transitions			Character
1	E = 3.62 eV	λ = 241 nm	f = 0.04	
	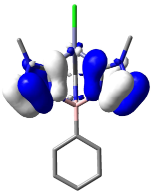	0.95 →	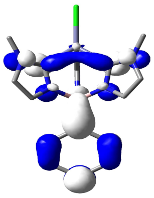	LC/LLCT
55	E = 3.67 eV	λ = 180 nm	f = 0.27	
	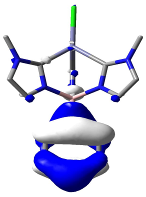	0.51 →	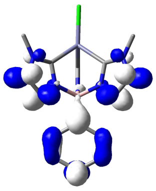	LC/LLCT
	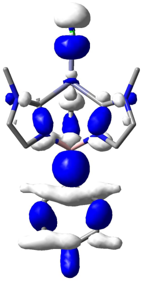	0.20 →	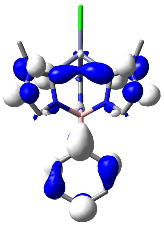	LC/LLCT
	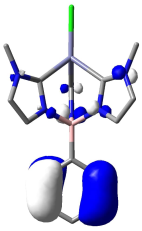	0.16 →	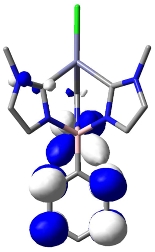	LC

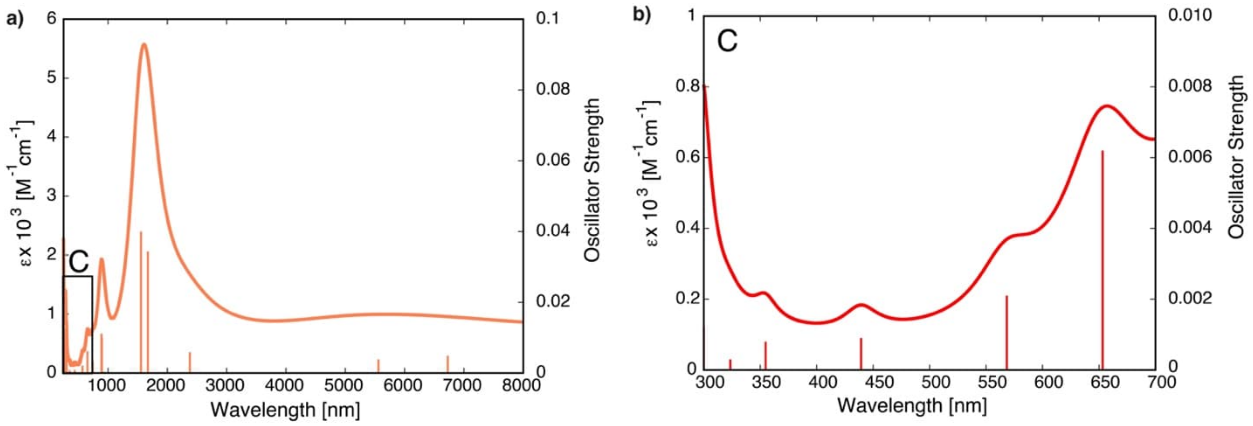

Figure S24. Calculated absorption spectrum with corresponding stick spectrum of the oxidized $[Zn^{II}(L)Cl]^{+}$.

Table S4. Natural transition orbital (NTO) analysis for the excited state transitions occurring at λ = 241 nm and 180 nm of the oxidized $[Zn^{II}(L)Cl]^{1+}$. (NTO coefficients are shown above the arrows)

Excited State	Hole-particle pair transitions	Character
12	E = 1.90 eV λ = 653 nm f = 0.006	LC
	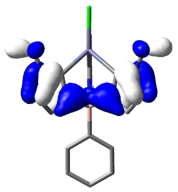 1.00 → 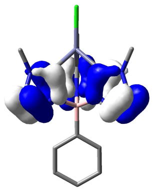	
13	E = 2.18 eV λ = 568 nm f = 0.002	LLCT
	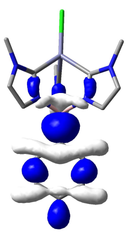 1.00 → 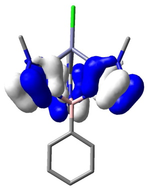	

Table S5. Final optimized complex geometry (starting point from the crystal structure coordinates of the XRD data), together with the main bond distances and angles.

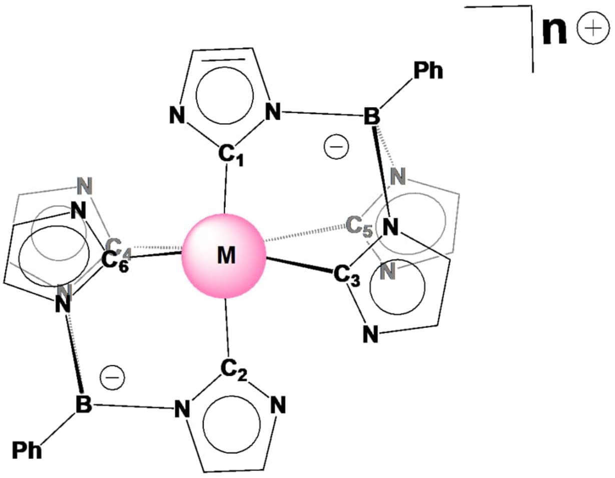

Geometrical Parameters	Experimental (Å)	Theoretical (Å)
Mn-C_{ax1}	2.023	2.042
Mn-C_{ax2}	2.024	2.042
Mn-C_{eq3}	2.053	2.071
Mn-C_{eq4}	2.052	2.071
Mn-C_{eq5}	2.052	2.070
Mn-C_{eq6}	2.051	2.070
C_{ax1}-Mn-C_{ax2}	179.99	180.00
C_{eq3}-Mn-C_{eq4}	179.96	180.00
C_{eq5}-Mn-C_{eq6}	179.98	180.00

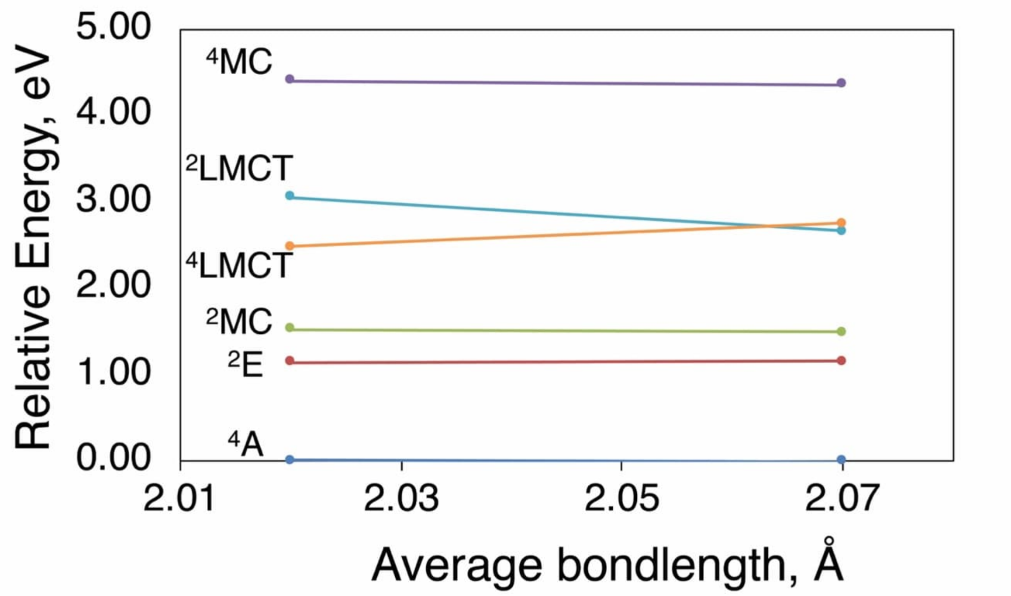

Figure S25. Potential energy surface for the relevant electronic states of $[Mn^{IV}L_2]^{2+}$ from energies obtained at the ^2E (2.02 Å) and ^4A (2.07 Å) optimized structures

6. References

(1) Kjær, K. S.; Kaul, N.; Prakash, O.; Chábera, P.; Rosemann, N. W.; Honarfar, A.; Gordivska, O.; Fredin, L. A.; Bergquist, K. E.; Häggström, L.; Ericsson, T.; Lindh, L.; Yartsev, A.; Styring, S.; Huang, P.; Uhlig, J.; Bendix, J.; Strand, D.; Sundström, V.; Persson, P.; Lomoth, R.; Wärnmark, K. Luminescence and Reactivity of a Charge-Transfer Excited Iron Complex with Nanosecond Lifetime. *Science* 2019, *363* (6424), 249–253. https://doi.org/10.1126/science.aau7160.

(2) Van Stokkum, I. H. M.; Larsen, D. S.; Van Grondelle, R. Global and Target Analysis of Time-Resolved Spectra. *Biochim Biophys Acta Bioenerg* 2004, *1657* (2–3), 82–104. https://doi.org/10.1016/j.bbabio.2004.04.011.

(3) Snellenburg, J. J.; Laptenok, S.; Seger, R.; Mullen, K. M.; van Stokkum, I. H. M. Glotaran: A Java-Based Graphical User Interface for the R Package TIMP. *Journal of Statistical Software;* 2012*, 49,* (3), 1-22.

(4) Yang, H.; Bard, A. J. The Application of Fast Scan Cyclic Voltammetry. Mechanistic Study of the Initial Stage of Electropolymerization of Aniline in Aqueous Solutions. *Journal of Electroanalytical Chemistry* 1992, *339* (1), 423–449. https://doi.org/https://doi.org/10.1016/0022-0728(92)80466-H.

(5) Müller, P.; Brettel, K. [Ru(Bpy)3]2+ as a Reference in Transient Absorption Spectroscopy: Differential Absorption Coefficients for Formation of the Long-Lived ^{3}MLCT Excited State. *Photochemical and Photobiological Sciences* 2012, *11* (4), 632–636. https://doi.org/10.1039/c2pp05333k.

(6) Kalyanasundaram, K. Luminescence and Redox Reactions of the Metal-to-Ligand Charge-Transfer Excited State of Tricarbonylchloro-(Polypyridyl)Rhenium(I) Complexes. *Journal of the Chemical Society, Faraday Transactions 2* 1986, *82* (12), 2401. https://doi.org/10.1039/f29868202401.

(7) Ilic, A.; Schwarz, J.; Johnson, C.; de Groot, L. H. M.; Kaufhold, S.; Lomoth, R.; Wärnmark, K. Photoredox Catalysis via Consecutive ^{2}LMCT- and ^{3}MLCT-Excitation of an Fe(III/II)–*N*-Heterocyclic Carbene Complex. *Chem Sci* 2022, *13* (32), 9165–9175. https://doi.org/10.1039/D2SC02122F.
